# Gestational age data completeness, quality and validity in population-based surveys: EN-INDEPTH study

**DOI:** 10.1186/s12963-020-00230-3

**Published:** 2021-02-08

**Authors:** M. Moinuddin Haider, Kaiser Mahmud, Hannah Blencowe, Tahmeed Ahmed, Joseph Akuze, Simon Cousens, Nafisa Delwar, Ane B. Fisker, Victoria Ponce Hardy, S. M. Tafsir Hasan, Md. Ali Imam, Dan Kajungu, Md Alfazal Khan, Justiniano S. D. Martins, Quamrun Nahar, Obed Ernest A. Nettey, Adane Kebede Tesega, Judith Yargawa, Nurul Alam, Joy E. Lawn, the Every Newborn-INDEPTH Study Collaborative Group, Peter Byass, Joy E. Lawn, Peter Waiswa, Hannah Blencowe, Judith Yargawa, Joseph Akuze, Ane B. Fisker, Justiniano S. D. Martins, Amabelia Rodrigues, Sanne M. Thysen, Gashaw Andargie Biks, Solomon Mokonnen Abebe, Tadesse Awoke Ayele, Telake Azale Bisetegn, Tadess Guadu Delele, Kassahun Alemu Gelaye, Bisrat Misganaw Geremew, Lemma Derseh Gezie, Tesfahun Melese, Mezgebu Yitayal Mengistu, Adane Kebede Tesega, Temesgen Azmeraw Yitayew, Simon Kasasa, Edward Galigawango, Collins Gyezaho, Judith Kaija, Dan Kajungu, Tryphena Nareeba, Davis Natukwatsa, Valerie Tusubira, Yeetey A. K. Enuameh, Kwaku P. Asante, Francis Dzabeng, Seeba Amenga Etego, Alexander A. Manu, Grace Manu, Obed Ernest Nettey, Sam K. Newton, Seth Owusu-Agyei, Charlotte Tawiah, Charles Zandoh, Nurul Alam, Nafisa Delwar, M. Moinuddin Haider, Md. Ali Imam, Kaiser Mahmud, Angela Baschieri, Simon Cousens, Vladimir S. Gordeev, Victoria Ponce Hardy, Doris Kwesiga, Kazuyo Machiyama

**Affiliations:** 1 Health Systems and Population Studies Division, icddr,b, Dhaka, Bangladesh; 2 Maternal, Adolescent, Reproductive & Child Health (MARCH) Centre, London School of Hygiene & Tropical Medicine, London, UK; 3 Nutrition and Clinical Services Division, icddr,b, Dhaka, Bangladesh; 4 Centre of Excellence for Maternal Newborn and Child Health Research, Makerere University, Kampala, Uganda; 5 Department of Health Policy, Planning and Management, Makerere University School of Public Health, Kampala, Uganda; 6 Centre for Statistical Methodology, London School of Hygiene & Tropical Medicine, London, UK; 7 Bandim Health Project, Bissau, Guinea-Bissau; 8 Research Centre for Vitamins and Vaccines, Statens Serum Institut, Copenhagen, Denmark; 9 Open Patient Data Explorative Network (OPEN), University of Southern Denmark, Odense, Denmark; 10 IgangaMayuge Health and Demographic Surveillance System, Makerere University Centre for Health and Population Research, Makerere, Uganda; 11 Matlab Health Research Centre, icddr,b, Dhaka, Bangladesh; 12 Kintampo Health Research Centre, Kintampo, Ghana; 13 Dabat Research Centre Health and Demographic Surveillance System, Dabat, Ethiopia; 14 Department of Health Systems and Policy, University of Gondar Institute of Public Health, Gondar, Ethiopia

**Keywords:** Gestational age, Preterm birth, Household survey, Demography, Surveillance, Last menstrual period, Ultrasound

## Abstract

**Background:**

Preterm birth (gestational age (GA) <37 weeks) is the leading cause of child mortality worldwide. However, GA is rarely assessed in population-based surveys, the major data source in low/middle-income countries. We examined the performance of new questions to measure GA in household surveys, a subset of which had linked early pregnancy ultrasound GA data.

**Methods:**

The EN-INDEPTH population-based survey of 69,176 women was undertaken (2017-2018) in five Health and Demographic Surveillance System sites in Bangladesh, Ethiopia, Ghana, Guinea-Bissau and Uganda. We included questions regarding GA in months (GAm) for all women and GA in weeks (GAw) for a subset; we also asked if the baby was ‘born before expected’ to estimate preterm birth rates. Survey data were linked to surveillance data in two sites, and to ultrasound pregnancy dating at <24 weeks in one site. We assessed completeness and quality of reported GA. We examined the validity of estimated preterm birth rates by sensitivity and specificity, over/under-reporting of GAw in survey compared to ultrasound by multinomial logistic regression, and explored perceptions about GA and barriers and enablers to its reporting using focus group discussions (*n* = 29).

**Results:**

GAm questions were almost universally answered, but heaping on 9 months resulted in underestimation of preterm birth rates. Preference for reporting GAw in even numbers was evident, resulting in heaping at 36 weeks; hence, over-estimating preterm birth rates, except in Matlab where the peak was at 38 weeks. Questions regarding ‘born before expected’ were answered but gave implausibly low preterm birth rates in most sites. Applying ultrasound as the gold standard in Matlab site, sensitivity of survey-GAw for detecting preterm birth (GAw <37) was 60% and specificity was 93%. Focus group findings suggest that women perceive GA to be important, but usually counted in months. Antenatal care attendance, women’s education and health cards may improve reporting.

**Conclusions:**

This is the first published study assessing GA reporting in surveys, compared with the gold standard of ultrasound. Reporting GAw within 5 years’ recall is feasible with high completeness, but accuracy is affected by heaping. Compared to ultrasound-GAw, results are reasonably specific, but sensitivity needs to be improved. We propose revised questions based on the study findings for further testing and validation in settings where pregnancy ultrasound data and/or last menstrual period dates/GA recorded in pregnancy are available. Specific training of interviewers is recommended.

## Key findings

**Table:** 

**WHAT IS NEW?**
• **What was known already:** Gestational age (GA) is instrumental in ascertaining foetal maturity and identifying preterm births; however, it is rarely assessed in population-based surveys. Quality of survey GA data, and barriers and enablers to GA data collection in such surveys have been unstudied. • **What was done:** Analyses of a population-based survey of 69,176 women of reproductive age including novel questions on GA to assess feasibility and quality (completeness, heaping), as well as acceptability (qualitative data) in five HDSS sites, plus validity against gold standard by early ultrasound in one site (Matlab, Bangladesh).
**WHAT WAS FOUND IN THE QUANTITATIVE DATA?**
• **Completeness:** GAm was reported for almost all births in all sites. Data on GAw was more variable. In four sites, interviewers prompted women leading to an estimate of GAw for 56-98% of births. In Bandim (Guinea-Bissau), where no prompting was used, only 6% were able to report GAw. • **Data quality (heaping):** In Matlab (Bangladesh), survey-reported GA in months and weeks yielded similar preterm birth rates. In the other four sites, reported GAm heaped at 9 months, underestimating preterm birth rate and GAw heaped at even numbers, particularly 36 weeks, overestimating preterm birth rate. • **Validity:** Compared to early pregnancy ultrasound, in Matlab (*n* = 481), the sensitivity of survey GAw was 60% with specificity of 93%. The sensitivity of HDSS-GAw, where date of last menstrual period was recorded in early pregnancy with an early pregnancy test was 66% and specificity was 95%.
**WHAT WAS FOUND IN THE QUALITATIVE DATA?**
• **Perceived value:** Women know the importance of tracking GA, notably for birth planning. Women count GA in months, not in weeks. Counting GAm from missed periods is common practice facilitated by religious and cultural events, crop harvesting times etc. • **Barriers/enablers:** Barriers to reporting GA include lack of awareness of menstrual cycles, not retaining health cards and fear of social stigma and witchcraft.
**WHAT NEXT IN MEASUREMENT AND RESEARCH?**
• **Measurement improvement now:** Whilst heaping may remain a challenge, we note that other variables such as birth weight are collected in surveys despite considerable heaping and missing data. More investment and innovation are warranted given the importance of GA data for estimating preterm birth rates and data gaps in the highest-burden settings. Based on the findings in this study, we propose a revised set of questions to collect GAw. • **Research needed:** Further studies to refine GA collection methods, link to card data and improve consistency in probing could lead to more robust approaches to assess GA in surveys. Innovation with dating apps and improving women's awareness of menstrual cycle dating are also key.

## Background

Preterm birth is the leading cause of child deaths worldwide, causing an estimated one million deaths per year and a high burden of morbidity for children and their families [1–3]. Each year, an estimated 15 million babies are born preterm, the majority (91%, 13.6 million) in low- and middle-income countries (LMICs) with over 80% in Asia and sub-Saharan Africa. Accurate and timely data on preterm birth are needed to inform appropriate resources and interventions and to monitor trends. The World Health Organization (WHO) has committed to providing updated estimates of preterm births every 3 to 5 years to support progress towards targets such as the Sustainable Development Goals and the Every Newborn Action Plan, aiming to end preventable neonatal deaths and stillbirths by 2030 [1, 2]. However, substantial gaps remain in the data, especially from the highest-burden settings.

WHO defines preterm birth as any birth before 37 completed weeks of gestation as measured from the 1st day of the last menstrual period (LMP) (Table 1) [4, 5]. Measurement of gestational age (GA) is essential for identifying preterm births [10, 11]. The ‘gold standard’ measure of GA is to assess the baby’s crown-rump length by ultrasound during early pregnancy (<14 weeks). Accuracy of ultrasound scan before 24 weeks is also considered acceptable since the difference in ultrasound-GAs measured between ≤13 weeks and 14-≤ 23 weeks is less than 1 week and falls with 95% confidence interval [12, 13]. Ultrasound measures at later gestations are less accurate [14]. However, in countries with the highest burden of preterm births, the timing of the first antenatal care (ANC) visit is typically in the second trimester and access to ultrasound is limited [15]. Hence, GA is commonly assessed from the date of the last menstrual period (LMP) [10]. This method has the advantage that it can be measured at any point during pregnancy, but accuracy is highest when recorded early in pregnancy [16]. LMP has lower accuracy (± 2-3 weeks) when compared to early pregnancy ultrasound scans [16–23]. Additionally, lower socio-economic status, limited literacy, high parity, and younger age are associated with increased uncertainty regarding LMP [24]. Other commonly used surrogates for GA measurement are described in Table 1 [21, 25, 26].

**Table 1:** Overview of definitions and measurements relating to gestational age

Gestational age	Measure of pregnancy duration in weeks, from the first day of the woman’s last menstrual cycle to the date of assessment
**Preterm birth**	Preterm birth is defined by WHO as ‘any birth before 37 completed weeks of gestation, or fewer than 259 days since the first day of the women’s last menstrual period (LMP)’ [4, 5]. It is subdivided into extremely preterm (< 28 weeks); very preterm (28-< 32 weeks) and moderate or late preterm (32-< 37 weeks) [6].
**Post-term birth**	Post-term birth is defined by WHO as ‘any birth at/after 42 completed weeks of gestation since the first day of the women’s last menstrual period (LMP)’ [4, 5].
**Measuring gestational age before birth**	*Ultrasound* (*gold standard*): Accurate to ± 5 to ± 21 days depending on the timing of ultrasound at different stage of pregnancy and biometric parameters (crown-rump length, biparietal diameter, femur length, abdominal circumference, head circumference). CRL was used for pregnancy of < 14 weeks, and BPD and FL were used for ≥ 14 to < 24 weeks. *Last menstrual period* (*LMP*): Women’s recall of the date of the first day of her last period. Accurate to ~ ± 14-21 days. Affected by variation in menstrual cycles (e.g. with under-nutrition or after cessation of hormonal contraceptive methods, or breastfeeding), socio-cultural attitudes to menstruation, literacy levels and digit preference. *Best obstetric measure*: Combination of ultrasound and LMP. *Fundal height*: Symphysis-fundal height measurement is another method that can estimate gestational age before birth. WHO guideline development group does not recommend this method due to lack of evidence in accuracy [7].
**Measuring gestational age after birth**	There are a range of clinical assessments, e.g. Dubowitz, Ballard, Finnstrom scores and others [8, 9]. This study does not apply these methods.

Measurement of child health and pregnancy outcomes in high burden countries which account for around two-thirds of the world’s births still rely mainly on large-scale household surveys like Demographic and Health Surveys (DHS) rather than on civil and vital registration or routine health management information systems (HMIS) [11]. Most surveys, including DHS, do not include questions on GA for livebirths. However, questions which use women’s report of GA are asked for non-livebirths in DHS to classify stillbirths, and for neonatal deaths in verbal autopsy tools [27, 28].

To our knowledge, no study has so far assessed GA questions to add to a survey such as DHS, and compared these against a gold standard early pregnancy ‘ultrasound measurement’.

This paper is part of a series of papers from the Every Newborn-International Network for the Demographic Evaluation of Populations and their Health (EN-INDEPTH) study in five health and demographic surveillance system (HDSS) sites in Africa and Asia. This paper addresses three objectives:

1. *Investigate completeness and feasibility of recording GA* data in months and weeks by women’s report in the EN-INDEPTH population-based survey in five HDSS sites using new/modified questions, including predictors of reporting.
2. *Compare accuracy of GA reported in the EN-INDEPTH survey* to GA recorded through prospective health and demographic surveillance (Bandim and Matlab sites) and to GA assessed through early pregnancy ultrasound (Matlab site).
3. *Undertake qualitative research to assess community perceptions, practices and barriers to reporting GA in population-based surveys*, and identify commonalities and differences across the sites

## Methods

### EN-INDEPTH study design and settings

**Fig. 1:**
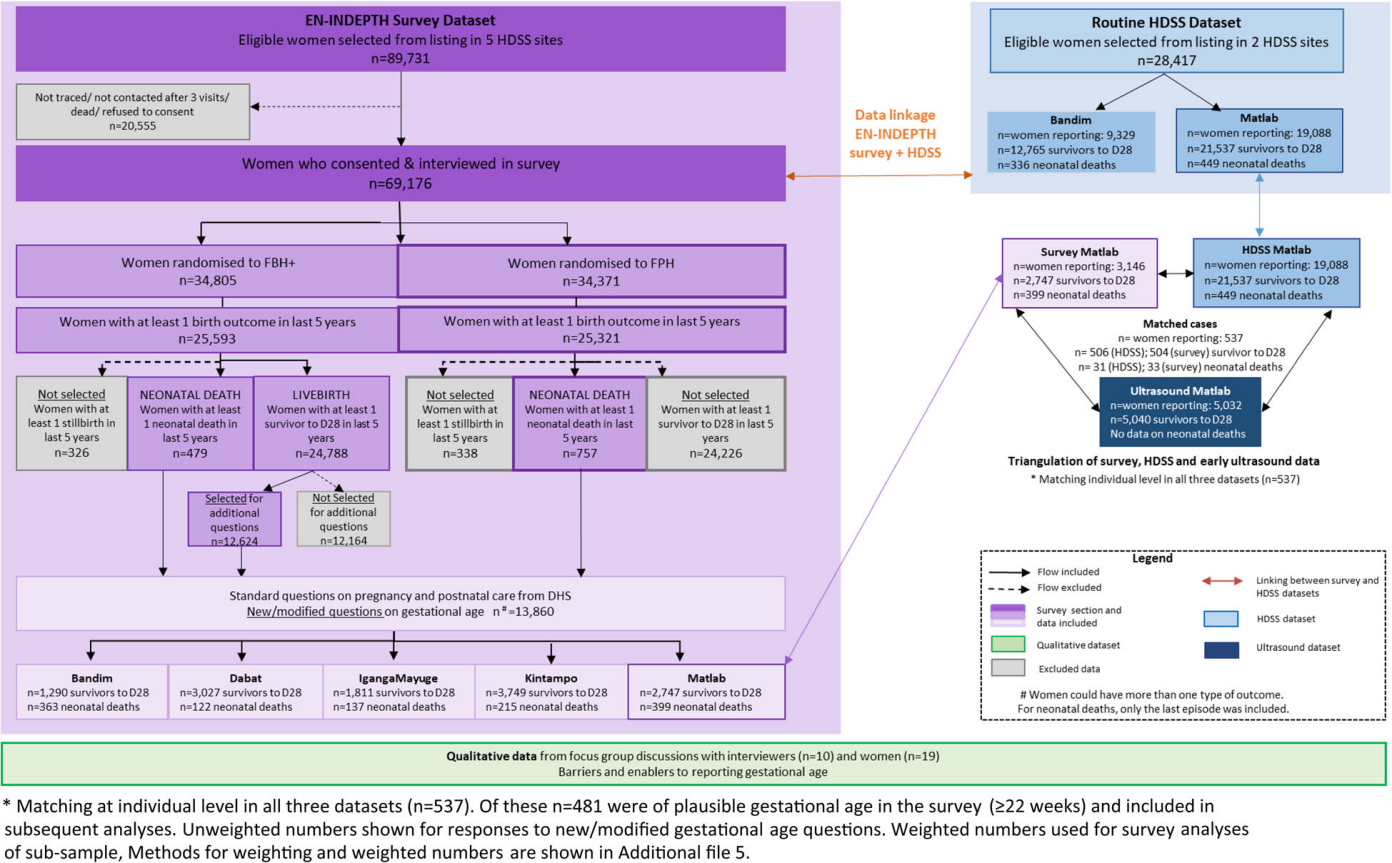
Flow diagram of EN-INDEPTH study population showing data included for gestational age analyses

The EN-INDEPTH study was a cross-sectional multi-site study conducted between July 2017 and August 2018, including a survey of 69,176 women aged 15-49 years in five HDSS sites: Bandim in Guinea-Bissau, Dabat in Ethiopia, IgangaMayuge in Uganda, Matlab in Bangladesh and Kintampo in Ghana (Fig. 1). The protocol and main study paper are published elsewhere and provide further details [29, 30]. The primary objective of the study was to compare two methods of retrospective recording of pregnancy outcomes in surveys: full birth history with additional questions on pregnancy losses (FBH+), and full pregnancy history (FPH) as detailed elsewhere [29, 30].

Both woman and interviewer data were collected on Android tablets using the Survey Solutions data collection and management system [31]. Interviewers were recruited locally and were familiar with the culture and dialect of the study area. Following completion of data collection, data from the five HDSS sites were anonymised by local HDSS scientists, encrypted and then shared [29]. Data management and analysis were done using Stata version 15.1. Results are reported in accordance with STROBE Statement checklists for cross-sectional studies [32] (Additional file 1).

Focus group discussions (FGDs) with survey respondents and interviewers, and a survey of interviewers were performed in March-August 2018 [33]. Information on perceptions, practices, and barriers relating to knowledge and reporting of GA was collected. Qualitative data were transcribed using a combination of notes and audio recordings, and were coded and analysed using the qualitative data analysis software, NVivo 12.

### Survey questions and HDSS linkage for gestational age

The EN-INDEPTH study also investigated the performance of existing or modified survey questions to capture other pregnancy-related outcomes including GA (Table 2). GA reported in months (GAm) was collected for all livebirths in the 5 years preceding the EN-INDEPTH survey. A sub-sample of survey respondents in all sites were also asked to report GA in weeks (GAw), and if they were ‘born before expected’ the number of weeks early for their most recent surviving livebirth, and all neonatal deaths in the last 5 years (Additional file 2). The two-part question on the woman’s perspective of whether her baby was ‘born before expected’, was adapted from the 2007 version of WHO’s Verbal Autopsy tool [28]. GAw was collected from health cards where available, or from recall. GAm and number of weeks early was collected by recall only. For babies reported to be ‘born before expected’, GA in weeks was estimated as 40 minus the number of weeks early. A livebirth of GA < 9 months or GA < 37 weeks was coded as a preterm birth. Livebirths with reported GA ≤ 5 months or GA ≤ 21 weeks were excluded as survival below these limits is biologically implausible.

**Table 2:** EN-INDEPTH survey questions for gestational age and method of administering these questions

Standard questions in DHS-7		New/modified questions asked on GA in EN-INDEPTH		
***Question***	***Details***	***Question***	***Details***	***Probing, calculation at data collector’s end, and recording***
‘How many months pregnant were you when that pregnancy ended?’	Only asked for non-livebirths. Asked in additional questions on non-livebirths after the roster (section 2).	‘How many months did this pregnancy last?’	Asked for all births and pregnancy losses in FBH+ and FPH in roster. Acceptable range 0-11. Red error message shown if value outside range entered.	- Probed when women could not spontaneously report. - GA was recorded in completed months, e.g. a reported GA of 9 months 2 weeks was recorded as 9 months.
		‘How many weeks pregnant were you when xxx was born?’	Asked for all last neonatal deaths and stillbirths since the 1st Jan 2012, and subset of surviving livebirths in section 4. Acceptable range 0-45. Red error message shown if value outside range entered. Data were collected from health cards (ANC card, maternal card or others) where it was available to women, else from recall.	- Most women reported GA in months. Then they were asked whether it was exactly the reported complete months, or the pregnancy lasted few more days/weeks, or that ended few days/weeks before the reported month. - Women’s reported GAs in months were converted into weeks and recorded in completed weeks at interview. - Months and fraction of months (in days/weeks) reported after complete months were converted to weeks considering 30 days month in Matlab. - Bandim site did not probe. When women replied ‘don’t know’ the response was recorded as such.
		‘Was xxx born before expected?	Asked for all last neonatal deaths and stillbirths since the 1st Jan 2012 and subset of surviving livebirths in section 4. Responses coded—Yes/No/Don’t Know. Those responding ‘Yes’ were asked: ‘How many weeks was xxx born before the expected date of delivery?’ Response—numeric integer. Option to report in months if weeks are not known.	- Probed when women could not spontaneously report - Recorded in completed weeks - Recorded ‘0’ for < 7 days - For months, recorded as the women reported

The EN-INDEPTH survey data were linked with HDSS data in the two sites where dates of LMP (Matlab, Bangladesh), and reported months of pregnancy at pregnancy registration (Bandim, Guinea-Bissau) were routinely recorded along with pregnancy outcomes (Additional file 3). In Matlab, ultrasound data from icddr,b Matlab Hospital (Additional file 3) were also linked [29]. For these two sites, individual pregnancy records included in the EN-INDEPTH study since the 1st January 2012 were matched with that in the HDSS records using probabilistic matching (Additional file 4). Matlab Hospital records HDSS IDs in clinical records, enabling the matching of the ultrasound report with HDSS records. After probabilistic linking of births captured in survey with births in the HDSS, the matched children’s HDSS IDs were used to match ultrasound records. Only early ultrasound pregnancy dating reports at < 24 weeks were included in GA analyses [12].

## Data analyses

### *Objective 1: completeness and feasibility of recording GA data in population-based surveys*

For analyses of GAw and ‘born before expected’ questions, sample weights were applied using the *svyset* command to account for the different probability of a neonatal death being included compared to a livebirth surviving the neonatal period, given that women’s response may vary for these two groups (Additional file 5). Descriptive statistics were used to analyse responses (any/plausible response) and digit/number preference for GA questions. Logistic regression was used to examine evidence of variations in GAw reporting (reporting any value against not reporting or reporting ‘don’t know’) by socio-demographic characteristics and maternal care-seeking behaviour. Preterm birth rates were calculated for each approach and compared to national estimates to assess plausibility of GA responses at a population level.

Century month code, DHS’s date data coding system that uses month and year, was used to identify events occurring in the 5 years prior to the interview. Socioeconomic wealth quintiles were used to measure the wealth status of households and were derived from infrastructure, housing and assets owned using Principle Components Analysis as used by DHS and MICS [34].

### *Objective 2: accuracy of survey reported GA compared to routine HDSS and ultrasound data*

GAw was calculated from HDSS data (Bandim and Matlab) and ultrasound data (Matlab only) (Additional file 3). In view of missing GAw in survey data from Bandim, survey GAm was compared with GAw from HDSS. In Matlab, GAw from the survey was compared to HDSS and ultrasound data (gold standard), and GAw from HDSS with ultrasound data. We categorized GAw in four groups (extreme and very preterm, 22 ≤ GAw ≤ 31; moderate preterm, 32 ≤ GAw ≤ 36; term and post-term, 37 ≤ GAw) and then compared the groups based on GA estimates from HDSS and survey with the groups based on GAw from ultrasound. Sensitivity and specificity of preterm birth detection by GAw from the survey and HDSS were assessed. Bland-Altman mean difference (MD) between sources with 95% limits of agreement, concordance correlation coefficients (CCC) with 95% confidence interval (CI), and kappa coefficients (KC) with 95% CI were used to assess agreement. We used multinomial logistic regression to examine over- and under-reporting of GAw in survey and HDSS compared to ultrasound.

### *Objective 3: qualitative research to assess barriers and enablers to survey reported GA*

To understand community perceptions and barriers related to GA reporting in household surveys, 29 focus group discussions (FGDs) were undertaken with 172 survey respondents and 82 survey interviewers and supervisors (Additional file 6) [33]. Thematic analysis to identify community perceptions, practices and barriers to reporting GA was conducted in NViVo 12 using an iterative process guided by an a priori codebook and addition of new codes that emerged during analysis. Themes were summarised and grouped to explore how findings contribute to understanding of the measurement of GA in population-based surveys.

## Results

### Overall

Information on GAm was collected for 65,562 livebirths in the last 5 years from 69,176 surveyed women. For the subsample of 13,860 livebirths, GAw and mother’s perception of whether the child was born before the expected date was also collected (weighted number 15,086) (Fig. 1). Survey respondents differed across HDSS with regards to age, parity, education and religion (Table 3).

**Table 3:** Characteristics of women with at least one birth outcome in the last 5 years, EN-INDEPTH survey (*n* = 50,914)

	Bandim	Dabat	IgangaMayuge	Kintampo	Matlab	All sites
**Number of women interviewed**	9109	6254	5632	11,411	18,508	50,914
**Age**						
15-19	4.8	3.8	6.0	2.6	5.2	4.5
20-24	22.6	18.2	21.7	15.1	27.9	22.2
25-29	26.6	25.7	24.9	21.7	29.8	26.3
30-34	23.2	21.0	20.7	24.1	23.6	23.0
35-39	15.2	17.8	14.7	20.4	10.7	15.0
40-44	6.1	9.7	8.9	11.6	2.5	6.8
45-49	1.7	3.7	3.2	4.5	0.3	2.2
Total	100%	100%	100%	100%	100%	100%
**Parity**						
1	27.1	17.3	19.0	16.6	32.2	24.5
2	23.1	16.9	16.0	18.0	35.8	25.0
3+	49.8	65.8	65.0	65.4	32.0	50.5
Total	100%	100%	100%	100%	100%	100%
**Education**						
Never attended school	29.1	61.6	8.4	39.3	4.4	24.1
Primary incomplete	43.5	17.8	48.7	22.8	11.3	24.6
Primary complete	13.6	3.7	9.7	5.1	8.7	8.2
Secondary incomplete	13.7	16.0	33.3	32.9	64.5	38.9
Secondary complete/above	0.1	0.9	0.0	0.0	11.2	4.2
Missing	0.0	0.0	0.0	0.0	0.0	0.0
Total	100%	100%	100%	100%	100%	100%
**Wealth index**						
Lowest	20.2	26.5	25.3	20.3	20.4	21.6
Second	20.1	19.3	21.6	20.0	19.9	20.1
Middle	20.0	20.8	18.7	20.0	19.8	19.9
Fourth	19.4	17.8	18.7	19.9	20.1	19.5
Highest	20.3	15.6	15.8	19.9	19.9	19.0
Total	100%	100%	100%	100%	100%	100%
**Religion**						
Muslim	39.5	3.3	57.0	31.8	88.4	53.0
Christian	40.5	96.7	42.9	62.3	0.0	37.8
Other or none	20.0	0.0	0.2	5.9	11.6	9.1
Missing	0.1	0.0	0.0	0.0	0.0	0.0
Total	100%	100%	100%	100%	100%	100%

### Objective 1: completeness and feasibility of recording GA data in population-based surveys

#### Completeness and plausibility of GA data captured in months

**Fig. 2:**
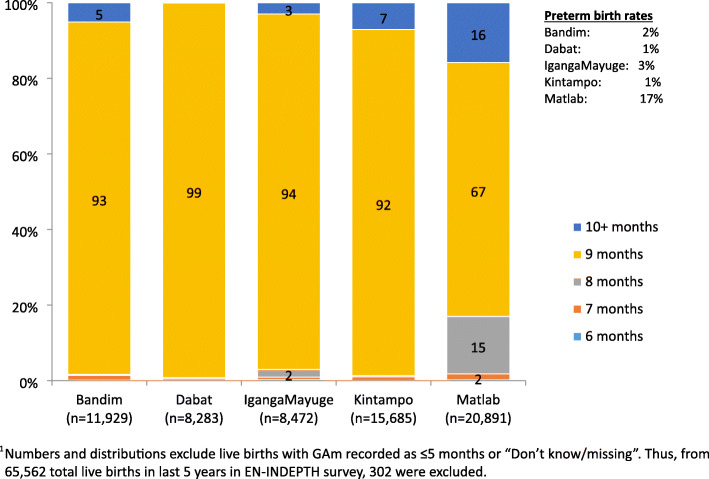
Distribution of reported gestational age in months by HDSS site, EN-INDEPTH survey (five sites, *n* = 65,260)^1^

Table 4 panel A shows near-universal reporting of GAm for livebirths in the last 5 years in all five sites. However, in all sites except Matlab, 91-99% of babies were reported to have been born at 9 months (Fig. 2).

**Table 4:** GA in months, weeks and ‘born before expected’ in the last 5 years, EN-INDEPTH survey

	HDSS sites					All sites
	Bandim	Dabat	IgangaMayuge	Kintampo	Matlab	
Number of women	9109	6254	5632	11,411	18,508	50,914
**Panel A: Gestational age in months for livebirths in the last 5 years preceding start of survey**						
Number of livebirths in the last 5 years	12,002	8295	8508	15,758	20,999	65,562
Implausible (≤ 5 months)	0.2	0.1	0.4	0.3	0.4	0.3
Plausible (≥ 6 months)	99.4	99.9	99.6	99.5	99.5	99.5
Do not know/missing	0.4	0.0	0.0	0.2	0.2	0.2
Total	100%	100%	100%	100%	100%	100%
Preterm birth rate^a^ (CI)	1.7 (1.5, 1.9)	0.8 (0.6, 1.0)	3.0 (2.7, 3.4)	1.4 (1.2, 1.6)	17.0 (16.5, 17.5)	8.0 (6.4, 6.8)
**Panel B: Gestational age in weeks for last livebirths in the last 5 preceding start of survey**						
Number of last livebirth in sub-sample (weighted)	2817	1978	2011	3629	4650	15,086
Implausible (≤ 21 weeks)	0.4	0.8	1.4	0.8	9.8	3.6
Plausible (≥ 22 weeks)	5.1	91.4	96.6	50.7	89.8	65.7
Do not know/missing	94.5	7.8	2.0	48.5	0.3	30.7
Total	100%	100%	100%	100%	100%	100%
Preterm birth rate^a^ (CI)	59.5 (50.5, 67.1)	96.6 (95.6, 97.4)	69.4 (67.3, 71.4)	71.5 (69.4, 73.6)	20.9 (19.7, 22.2)	54.2 (53.2, 55.2)
**Panel C: Babies ‘born before expected’ for livebirths in the last 5 years preceding start of survey**^**b**^						
Number of last livebirth in sub-sample (weighted)	2817	1978	2011	3629	4650	15,086
Implausible (≤ 21 weeks)	0.3	0.1	1.2	0.2	0.1	0.3
Plausible (≥ 22 weeks)	88.3	98.6	98.4	95.6	98.8	96.0
Do not know/missing whether the baby was born before expected	9.8	1.2	0.3	3.8	0.4	3.1
The baby was born before expected but do not know how many weeks before expected	1.6	0.0	0.2	0.4	0.4	0.6
Total	100%	100%	100%	100%	100%	100%
Preterm birth rate^c^ (CI)	1.0 (0.7-1.5)	0.8 (0.5-1.3)	2.8 (2.1-3.6)	1.7 (1.3-2.2)	7.7 (6.9, 8.5)	3.5 (3.2-3.8)

^a^Preterm birth rate was estimated amongst those who provided valid GA: GAm ≥ 6 and GAw ≥ 22

^b^GAw was measured by subtracting number of weeks the child was ‘born before expected’ from 40 weeks. The survey did not ask when the baby was expected to be born. We have assumed 40 weeks for all babies

^c^Preterm birth rate: GAw ≤ 36, like panel B. All ‘Don’t know/missing’ are excluded from the estimation

#### Completeness and plausibility of GA data captured in weeks

Completeness of GAw data was highly variable across sites (Table 4: panel B). In IgangaMayuge, 98.0% of women reported GAw compared to just 5.5% in Bandim. There were also reporting variations in GA in weeks by background characteristics (Additional file 7.1A). Reporting of GAw was higher amongst women above 30 years in Bandim; lower in women with ≥ 3 parity in Bandim and >3 parity in IgangaMayuge; higher in women who had ever attended school in Matlab; lower in highest wealth quintile in Bandim, in highest two wealth quintiles in Dabat and second to fourth wealth quintiles in Kintampo; lower in women affiliated with religions other than Islam and Christianity; lower amongst women who received 4+ ANC in Dabat and higher in 4+ ANC-receiving women in IgangaMayuge. Variations were not found by place of delivery.

Amongst those who reported to GAw, nearly all women reported a plausible value (GAw ≥ 22) in all sites except Matlab (Table 4: panel B). In Matlab, 9.8% of the births reported GAw ≤21 weeks including identical GAm and GAw for 8%. Half of the 8% was reported by one interviewer who recorded the same values for GAw and GAm in 160 out of 171 records. Another 1.6% was reported by two interviewers and 2.4% by the other 17 interviewers. Subsequent analyses excluded births with GAw ≤ 21.

**Fig. 3:**
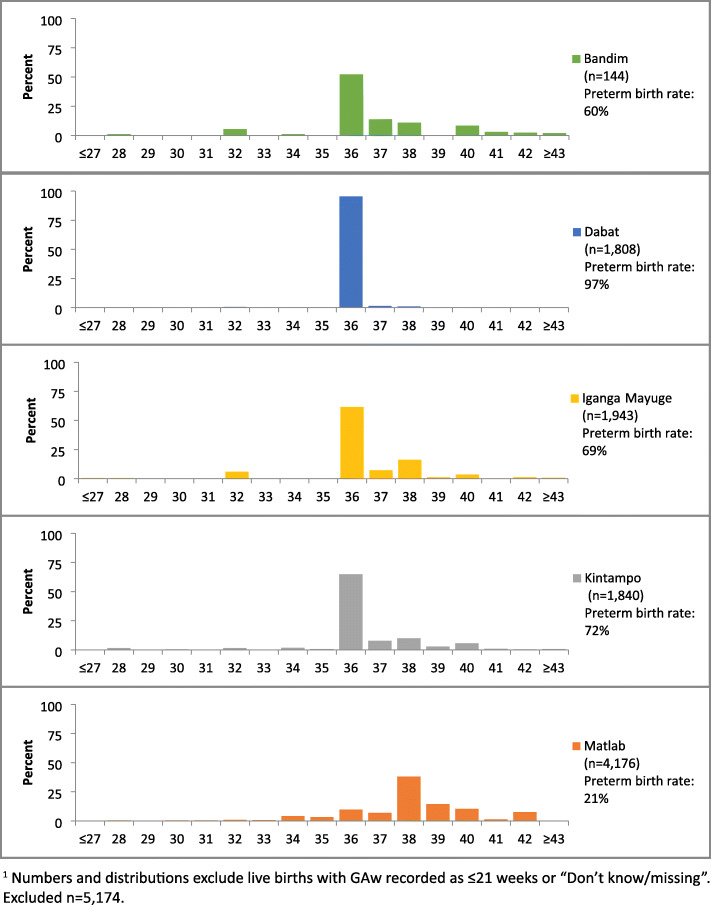
Distribution of reported gestational age in weeks by HDSS site, EN-INDEPTH survey (fives sites weighted, *n* = 9912)^1^

**Fig. 4:**
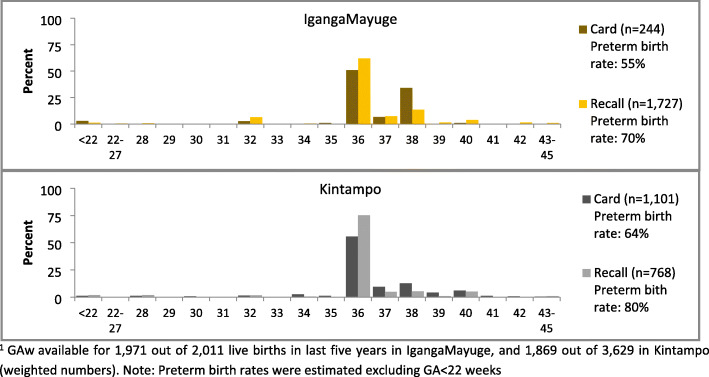
Comparison of GA weeks from card and recall, EN-INDEPTH survey, IgangaMayuge (*n* = 1971) and Kintampo (*n* = 1869)^1^

Few livebirths were reported at < 36 weeks in any site (Fig. 3). In all sites, a preference for even digits was observed, with GAw heaped at 36 weeks (equalling to 4 × 9 months, the most commonly recorded value for GAm) in four sites (Additional file 7.1B). The questions on GAw were designed to collect GAw from card (ANC/other health cards) where it was available, else from women’s recall. Of the GAw collected in the survey, 52% in Kintampo, 13% in IgangaMayuge and 0% in the other sites were from cards. Of the GAw from cards, <2% were ≤21 weeks. Greater variation in reported GAw was seen by card compared to recall. A higher proportion of births were reported at 38 weeks in both sites, and fewer births reported at 36 weeks in Kintampo by card compared to recall (Fig. 4).

#### Other questions regarding preterm birth

Over 96.3% of women answered the question ‘was xxx born before expected?’ (Table 4: panel C). The proportion of ‘Don’t know/missing’ responses to whether the baby was ‘born before expected’ or ‘Don’t know’ to how many weeks the baby was ‘born before expected’ was 9.8% in Bandim and below 4% in other sites. The proportion of babies reported to have been ‘born before expected’ was 28.3% in Matlab, but only 1.4-5.4% in other sites.

#### Estimated preterm birth rates based on the three survey approaches tested

The estimated preterm birth rate using GAm was 17.0% in Matlab, compared with ≤3% in all other sites (Table 4: panel A). GAw showed a similar preterm birth rate in Matlab (20.9%) but high rates in other sites (Dabat, 96.6%; Bandim, IgangaMayuge and Kintampo, 59.5-71.5%) (Table 4: panel B). The question ‘was xxx born before expected?’ provided lower preterm birth rates GAm and GAw in Matlab (7.7%), and similar rates to GAm in the other sites (0.8-2.8%) (Table 4: panel C). Preterm birth estimates from all three survey approaches tested were very different from national estimates in all sites apart from Matlab (Additional file 7.4).

### Objective 2: accuracy of survey reported GA compared to routine HDSS and ultrasound data

As only 5.7% of livebirths reported survey-GAw, estimated HDSS-GAw were compared to survey-GAm. HDSS-GAw was available for 5725 livebirths out of 13,456 livebirths with GAm ≥6 in the survey. Estimated GAw in the HDSS is almost normally distributed with an estimated preterm birth rate of 30.9%. In total, 93.2% of reported GAm were heaped at 9 months with 5.3% at 10 months, and a very low estimated preterm birth rate of 1.3% (Fig. 5a).

**Fig. 5A:**
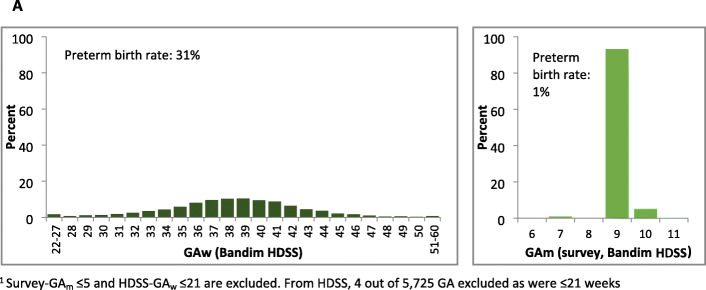
EN-INDEPTH survey GA weeks versus HDSS data GA months in the last 5 years, Bandim (*n* = 5721)^1^

In Matlab, data from 2776 of 2907 with GA ≥22 weeks in the EN-INDEPTH survey were matched with HDSS data. Figure 5b shows the GAw distribution where HDSS-GAw peaked at 39-40 weeks and survey-GAw at 38 weeks. The estimated preterm birth rate was 12.9% by HDSS-GAw and 22.1% by survey-GAw. A total of 1079 of 2907 livebirths in the survey were matched to ultrasound estimated GA, 542 of these were excluded as occurred at ≥ 24 weeks. The 537 ultrasound reports before 24 weeks were matched to HDSS and survey data. The quality of GA data for these 537 cases is shown in Fig. 6. Subsequent analyses include only the 481 livebirths with GA ≥ 22 weeks in the survey (Fig. 5c). HDSS-GAw had a similar number of livebirths reported at 38, 39 and 40 weeks. Ultrasound GAw peaked at 39 weeks. HDSS-GAw estimated more after 39 weeks and less before 37 weeks than ultrasound GAw. This resulted in a slightly lower estimated preterm birth rate in the HDSS (12%) than ultrasound (14%) (Fig. 5c). The survey GAw tended to heap on even numbers. Heaping on 36 weeks may explain the higher estimated preterm birth rates with survey GAw compared to HDSS GAw and ultrasound GAw (see Additional files 7.2A-7.2C).

**Fig. 5B:**
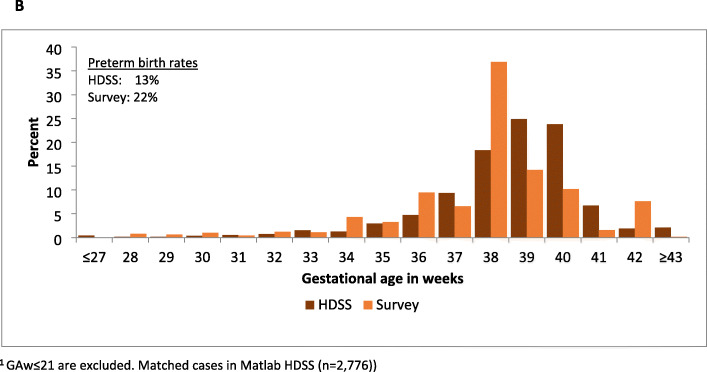
EN-INDEPTH survey versus HDSS data for GA weeks in the 5 years, Matlab (*n* = 2776)^1^

**Fig. 5C:**
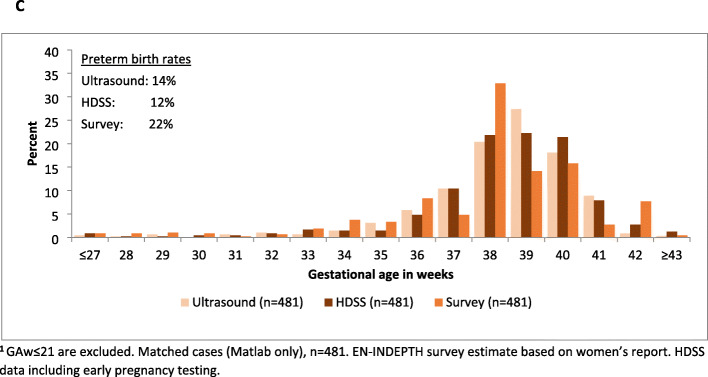
GA weeks (early pregnancy ultrasound) versus EN-INDEPTH survey, and HDSS data, last 5 years, Matlab (*n* = 481)^1^

**Fig. 6:**
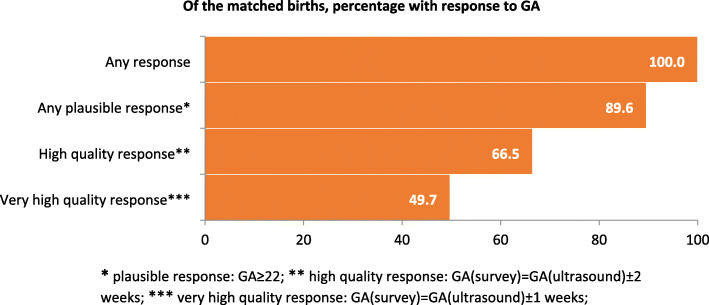
Data quality cascade for GA data, EN-INDEPTH survey matched with early pregnancy ultrasound, Matlab (*n* = 537)

**Fig. 7:**
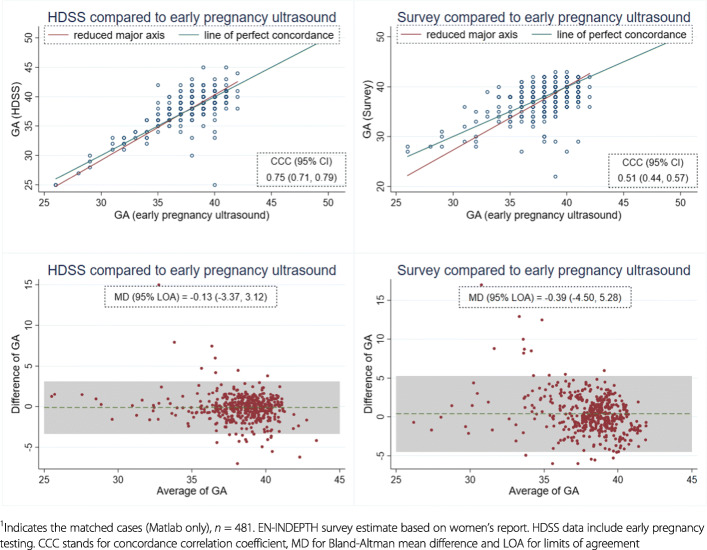
GA weeks comparing early pregnancy ultrasound with EN-INDEPTH survey and with HDSS (*n* = 481)^1^

Agreement by simple group-to-group matching of categorical data (extreme/very preterm, moderately preterm, term and post-term) between HDSS GAw and ultrasound GAw was 87.3%, and 71.5% between survey GAw and ultrasound GAw (Table 5A). However, the overall agreement between ultrasound GAw and HDSS GAw was weak (kappa coefficient (KC) = 0.54), and was poor between ultrasound GAw and survey GAw (KC = 0.25) [35]. For a simpler grouping (term and preterm), the agreement improved to 0.65 (KC) between HDSS-GAw and ultrasound-GAw, and to 0.36 (KC) between survey-GAw and ultrasound-GAw. Bland-Altman mean difference (MD) and concordance correlation coefficients showed similar results with better agreement between ultrasound GAw and HDSS GAw than ultrasound GAw and survey GAw (Fig. 7).

**Table 5A:** Agreement for gestational age category between ultrasound, HDSS and survey (Matlab only, *n* = 481)

	Extreme/very preterm^**a**^	Moderate preterm^**b**^	Term^**c**^	Post-term^**d**^	***n***	Kappa [confidence interval]
**Panel A: % of maturity group in HDSS matched with that of ultrasound (group-to-group match: 87.3%)**						
HDSS	Ultrasound					0.54 [0.49, 0.54]
Extreme/very preterm	**7**	1	2	0		
Moderate preterm	2	**34**	13	0		
Term	0	22	**378**	3		
Post-term	0	1	17	**1**		
*n*	9	58	410	4	481	
**Panel B: % of maturity-group in survey matched with that of ultrasound (group-to-group match: 71.5%)**						
Survey	Ultrasound					0.25 [0.22, 0.30]
Extreme/very preterm	**7**	4	7	0		
Moderate preterm	2	**27**	56	1		
Term	0	27	**309**	2		
Post-term	0	0	38	**1**		
*n*	9	58	410	4	481	

^a^22 ≤ GAw ≤ 31

^b^32 ≤ GAw ≤ 36

^c^37 ≤ GAw ≤ 41

^d^GAw ≥ 42

#### Validity of GA data in HDSS and survey compared to gold standard ultrasound GA data in Matlab

Of the GAw linked amongst ultrasound, HDSS and survey, 38.3% in HDSS and 20.4% in the survey had an exact match to ultrasound. Over reporting of GAw in both HDSS and survey was around one in three. Close to half (44.7%) of GAw in the survey were under reported.

Results from multinomial logistic regression did not find any variations in over- or under- reporting of GAw in the survey compared to ultrasound GAw by background characteristics. Lower over- reporting of HDSS GAw compared to ultrasound GAw was seen in the middle to fourth wealth quintiles, and higher over- reporting was observed in primary educated women. Higher under reporting was found in non-Muslims and primary educated women. Women’s age, parity, TV watching, ANC visits, place of delivery, icddr,b service area and survey recall period were not associated with over- or under- reporting (Additional file 7.2D).

The sensitivity of using HDSS collected GAw to detect preterm birth was 66%, and specificity was 95% compared to ultrasound ‘gold standard’ (Table 5B). Similar patterns with slightly lower levels were seen for survey collected GAw, with 60% sensitivity and 93% specificity.

**Table 5B:** Validity of GA weeks data comparing ultrasound to HDSS and to EN-INDEPTH survey (Matlab, *n* = 481)

**Panel A: Baby’s maturity—HDSS compared to ultrasound (gold standard)**					
		***Ultrasound***			Sensitivity (HDSS) = 65.7% Specificity (HDSS) = 94.5% Positive predictive value (HDSS) = 74.6% Negative predictive value (HDSS) = 96.4% Kappa [CI] = 0.65 [0.55, 0.76]
		**Preterm**^**a**^	**Term**^**b**^	*n*	
**HDSS**	**Preterm**	**44**	15	59	
	**Term**	23	**399**	422	
	*n*	67	414	481	
**Panel B: Baby’s maturity—survey compared to ultrasound (gold standard)**					
		***Ultrasound***			Sensitivity (survey) = 59.7% Specificity (survey) = 92.8% Positive predictive value (survey) = 38.5% Negative predictive value (survey) = 92.8% Kappa [CI] = 0.36 [0.26, 0.46]
		**Preterm**	**Term**	***n***	
**Survey**	**Preterm**	**40**	64	104	
	**Term**	27	**350**	377	
	***n***	67	414	481	

^a^22 ≤ GAw ≤ 36

^b^GAw ≥ 37

### Objective 3: qualitative research to assess barriers and enablers to survey reported GA

#### Community perceptions

Women perceived the importance of tracking the progress of pregnancy in all sites as this was seen to help in birth planning and preparation (Additional file 7.3). Facilitating fathers to be available to accompany the mother for ANC and delivery was another reason in IgangaMayuge. In Kintampo, women were scolded by healthcare providers if they could not report GA at ANC visits. Knowing the date of conception was also important in IgangaMayuge, especially for younger women to avoid denial of conception by the child’s biological father.

#### Community practices

Measuring or counting GA differed across sites. Women in Bandim found this difficult, whilst in IgangaMayuge it was perceived as easy. Women in IgangaMayuge and Kintampo reported that the ANC provider helped calculate GA.

Women tended to count GA in months in all sites. Missed periods, religious and cultural events, crop harvesting months and other key time points were used as reference points to count the months. Women in Dabat used key events in their religious calendar to recall their LMP date. For example, one woman stated, ‘my menstruation was terminated at Yetir Mariam’, meaning 21 January. In Matlab and Kintampo, women reported counting GA by missed periods—some counted their first missed period as the first month of GA, whilst a few others counted the first month of GA as their second missed period. GA counting in Matlab varied by religious affiliation. Hindu women counted 10 months 10 days for a full-term pregnancy whilst Muslim women counted 9 months.

*The Hindus usually tell like ten months ten days. In contrast the Muslims tell, ‘it remains nine months, does it exceed nine months?’ I worked mostly with the Hindus. I got ten months ten days from them, though I probed them well. Despite probing, they said ten months ten days. (Interviewer, Matlab, Bangladesh)*

#### Reported barriers and enablers

Women’s education (in Kintampo, Matlab and Dabat), and ANC attendance or facility birth (in Bandim) were perceived to improve GA reporting.

Barriers to knowing LMP included conceiving before their menses had returned following a previous pregnancy, cessation of hormonal contraceptives (Dabat and Matlab) or lack of awareness of menstrual cycles. Whilst health cards were perceived as a potential enabler, they were frequently poorly completed by healthcare workers and not preserved by many women. Social stigma and fear of witchcraft was an additional barrier to GA reporting in Bandim.

*Some don’t count their gestational days because of witchcraft; say, if SOMEONE else knows you are pregnant, he/she will be waiting for you at the birth on delivery day...sometimes someone is three or four months pregnant and still deny it and doesn't say anything. (Interviewer, Bandim, Guinea-Bissau)*

Some interviewers reported specific issues in obtaining GA information in Matlab and Kintampo sites where probes were required to help women recall LMP and GAm, and the interviewer then calculated GAw themselves based on information provided by the respondents.

## Discussion

Given the high burden of deaths and disability-adjusted life years due to preterm birth, improving data on gestational age is a high priority, especially from the highest-burden countries where household survey data remains a primary data source. To our knowledge, this is the first study to assess household survey questions on GA regarding feasibility, and importantly, validity compared with ultrasound-based GA as a gold standard in a subsample of the EN-INDEPTH study. Our findings in this large dataset from five countries suggest, whilst women can almost universally report GAm, these results are severely heaped on 9 months, with resultant underestimation of preterm birth rates. Reporting of GAw was feasible in Matlab, and these data were reasonably specific and of moderate sensitivity to detect preterm birth. In the other four sites, reporting of GAw was highly variable in terms of both completeness and quality of reported data. Further investment is needed to overcome the barriers to collecting data on GAw, and our study identifies some specific advances to improve the survey questions and the processes, underlining that addressing heaping is crucial.

GAm was very feasible to answer, with almost 100% of women responding but in four of the five sites severe heaping on 9 months resulted in implausibly low estimated preterm rates (<3%) (Additional file 7.4) [1, 36]. Such heaping might be the result of women’s rounding up to the month of delivery or rounding by the interviewer. The exception to this was the Matlab site where GA_m_ produced an estimated preterm birth rate of 17.0%.

Reporting of GAw was highly variable and required probing to obtain a specific response. Probing was not used in Bandim and 94% of responses were recorded as ‘don’t know or missing’, whilst in Kintampo, 44% of responses were ‘don’t know or missing’, even after probing. In four sites, it seems from the GAw distribution that GAw was predominantly calculated by the interviewers multiplying GAm by four, resulting in high estimated preterm birth rates (59.5-96.6%). In Matlab, data collectors were trained to multiply GAm by four and add 2 to get GAw, and to take into account any reported days or weeks before or after a completed month. This resulted in less heaping on 36 weeks, and an estimated preterm birth rate of 20.9%, which may still be an overestimate. Very few (<2%) of the reported GAw were implausible. Including in-built data quality checks for implausible responses, ≤ 21 weeks and implausible GAm/GAw combinations in future electronic data capture survey tools could reduce such errors. Further research is needed to test this approach in other settings.

A question to the woman, if her baby was ‘born before expected’, was adapted from the 2007 version of WHO’s Verbal Autopsy tool [28], and was feasible to answer but resulted in preterm birth rates which were implausibly low in all sites apart from in Matlab. Accurate answers require the woman to know her expected date of delivery (EDD). Whilst EDD should be routinely calculated at first ANC visit, despite 2/3rd ANC coverage in Dabat and > 90% in all other sites, these data suggest that this information is not communicated to the woman, or she is unable to recall or unwilling to report it.

Analyses of GA amongst the births in EN-INDEPTH survey linked with Matlab hospital’s pregnancy ultrasound data show similar rates of preterm births in ultrasound and HDSS, but higher in the survey. Other studies have found LMP-based measurement tends to report higher GA than ultrasound-based measurement [17, 22]. We note that GAw patterns and socio-demographic characteristics were similar between the groups, but ANC seeking and facility delivery were higher in matched group as the matched cases came from icddr,b service area. Over- or under- reporting of GAw in the survey compared to ultrasound were similar irrespective of women’s age, parity, TV watching, religion, dose-response of ANC care and place of delivery. All women amongst the matched group had at least one ANC in the last pregnancy, hence, status related to GA reporting remained unknown for women who did not receive any ANC. Survey GAw were over- reported amongst women with no education and under reported amongst women from second wealth quintile than ultrasound GAw.

Our qualitative data suggest that women track GA in pregnancy since this is perceived important to know to be able to plan for ANC and delivery, including getting the support of the father, and to be able to tell the health provider. Whilst almost all women in all sites were able to report GAm with plausible values, sometimes using religious and cultural events to assist recall, FGDs with women and interviewers suggested large variation in how women count ‘months’. Variation in reported length of gestation may be affected by cultural norms such as Matlab’s Hindu women reporting GA as 10 months 10 days, biological differences such variation in length of menstrual cycles or conceiving after a period of amenorrhoea, or use of different calendars such as 30.4 days in a Gregorian calendar compared to 29.5 days in a lunar calendar. All these can impact on comparability of survey-captured GA.

Improving GA data from population-based surveys requires that women know the information, and this may be facilitated by paper-based tools such as calendars, or smart-phone apps, improved access to early ANC and ultrasound and communication from health workers. Women must also be able and willing to report this information at the time of the survey. Including this information in ANC or maternal-child health cards could facilitate data availability at the time of the survey. In some settings, such as Bandim, social stigma and fear of witchcraft may need to be addressed.

Handheld health cards, such as antenatal or child health records are potentially effective for communicating information from health providers to an interviewer in a household survey, and are commonly used to collect information on birthweight [37, 38]. Although cards were expected to be better sources for GAw, we found similar GA distributions between cards and recall in Kintampo and IgangaMayuge. This may be as many women first attend ANC in late pregnancy and health workers, hence, rely on women’s reported LMP or stated GA. Health cards were rarely used to report GAw in other sites, which could be a missed opportunity, for example, in Bandim birthweight from card was available for 46.2% of livebirths compared to just 0.7% with GA from card [39]. GA-related information may vary by type of card and includes the expected date of delivery or GA at a visit or birth (in weeks or months). Processes used by interviewers to record GAw from the information on the card are unknown, and the higher than expected estimated preterm birth rates could be explained by conversion from months.

This study has strengths and limitations. Strengths include the large survey dataset from five LMICs, with consistent questions and analyses, plus multi-site comparable, qualitative data. Linkage of the survey with HDSS and ultrasound data from Matlab is novel, however, the generalisability of these findings may be limited as women with early ultrasound may be systematically different from other women in Matlab, for example with higher care-seeking, and from women in other settings without intensive pregnancy surveillance with widespread early pregnancy testing.

Access to ultrasound use during pregnancy is increasing, for example, in 2017, 74% of recently delivered women in Bangladesh reported having had an ultrasound during pregnancy [40]. However, early ultrasound coverage is presumed to be lower. In this study, only about a half of 1079 matched women with a pregnancy ultrasound in Matlab had the ultrasound before 24 weeks. In addition to the challenge in accessing care early in pregnancy, costs and infrastructure requirements may impede widespread early pregnancy ultrasound scale-up in many settings [41]. Where early ultrasound is not feasible, LMP may be reasonably accurate, especially in societies where cultural restrictions placed on the undertaking of certain activities increase awareness of menstrual cycles [42]. Innovative solutions are required to facilitate women’s full participation in society during menstruation, coupled with innovative methods to empower women to track their menstrual cycles. Prospective collection of LMP data alongside the use of a home calendar resulted in a high sensitivity (86%) and specificity (96%) for classifying preterm birth in Bangladesh [16].

Several of the challenges we identified regarding GA assessment in surveys are similar to those faced for birthweight in surveys, notably missing data, and heaping [38, 43]. Unlike GA, information on birthweight is routinely collected through household surveys and is sometimes used as a proxy for GA, although it is a poor proxy especially in South Asia where a high proportion of babies are born small for GA [3, 11]. In view of the importance of preterm and low birthweight outcomes, both GA and birthweight need further research to improve accuracy in survey data.

Based on these results, we propose a revised set of questions to collect GAw information retrospectively in household surveys (Additional file 8). These questions focus firstly on collating prospectively collected data to inform GA from ultrasound or ANC card, and only asking women’s retrospective report of length of gestation where no prospective data are available. These data could then be used by data collection apps during the survey or at the analysis stage standardise the calculation of GA.

## Conclusions

Estimates of preterm birth rates based on GA can be feasible from population-based surveys. However, more work is needed to improve the accuracy of reported GA and would be best focused on improving the capture of information on pregnancy duration in weeks, using prospectively collected data from early pregnancy ultrasound or ANC visit records where available. We propose revised questions, and standardised probes which can be tested against gold standard early ultrasound data for validation.

Given the value of GA data and the major global data gaps for preterm birth estimates, further investments and innovations are justifiable to improve GA data in surveys. Importantly, whilst accuracy may be improved by better survey tools, a pre-requisite is that women know their menstruation dates. This will require a shift in social norms, both to reduce the stigma in discussing menses and improving women's awareness regarding the recording of dates.

## Supplementary information

**Additional file 1.** STROBE guidelines checklist.

**Additional file 2.** Selection of women with a livebirth surviving the neonatal period, EN-INDEPTH survey.

**Additional file 3.** Overview of gestational age data collection in Matlab and Bandim HDSS sites.

**Additional file 4.** Linking between EN-INDEPTH survey and HDSS data.

**Additional file 5.** Calculation of survey weights. 5.1: Methods for calculation of survey weights. 5.2: Weighted numbers of livebirths by HDSS sites.

**Additional file 6.** Qualitative methods for Focus Group Discussions in the EN-INDEPTH study.

**Additional file 7.** Results: Additional details. 7.1: Gestational age capture in weeks in the EN-INDEPTH survey. 7.1A: Logit estimates of adjusted ORs and 95% confidence interval of responding to GA in weeks. 7.1B: Matching of GA months with GA weeks, EN-INDEPTH survey. 7.1C: Gestational age distribution by religion, Matlab site, EN-INDEPTH survey in last five years. 7.2: Comparison of GA weeks between survey, HDSS and early pregnancy ultrasound, Matlab site. 7.2A: GA weeks in last five years by HDSS, early pregnancy ultrasound and five years prior to EN-INDEPTH survey. 7.2B: GA weeks for livebirths by HDSS, early pregnancy ultrasound and EN-INDEPTH survey in last five years. 7.2C: Early pregnancy ultrasound versus EN-INDEPTH survey and HDSS data in last five years by ultrasound timing. 7.2D: Adjusted relative risk ratios for over- and under-reporting of GA weeks, survey and HDSS versus ultrasound. 7.3: Community perceptions, practices and barriers to reporting GA, EN-INDEPTH study (five sites). 7.4: Comparison of preterm birth rates in EN-INDEPTH study to external data sources.

**Additional file 8.** Proposed revised questions to capture gestational age.

**Additional file 9.** Ethical approval of local Institutional Review Boards.

## Data Availability

Data sharing and transfer agreements were jointly developed and signed by all collaborating partners. The datasets generated during the current study are deposited online at 10.17037/DATA.00001556 with data access subject to approval by collaborating parties.

## Abbreviations

CCC: Concordance correlation coefficient
CI: Confidence interval
DHS: Demographic and Health Survey
EN-INDEPTH: Every Newborn-International Network for the Demographic Evaluation of Populations and their Health
FBH+: Full birth history (+ denotes additional questions on pregnancy losses)
FGD(s): Focus group discussion(s)
FPH: Full pregnancy history
GA: Gestational age
GAm: Gestational age in months
GAw: Gestational age in weeks
HDSS: Health and demographic surveillance system
ID: Identification number
KC: Kappa coefficient
LMICs: Low- and middle-income countries
LMP: Last menstrual period
LOA: Limits of agreement
MD: Mean difference
MICS: Multiple Indicator Cluster Survey
WHO: World Health Organization
