# Supplementary material for: Gestational age data completeness, quality and validity in population-based surveys: EN-INDEPTH study
Source: Popul Health Metr. 2021 Feb 8;19(Suppl 1):16. doi: 10.1186/s12963-020-00230-3 (PMC7869446; doi:10.1186/s12963-020-00230-3)
Supplement: Supplementary file 3 — Additional file 3. Overview of gestational age data collection in Matlab and Bandim HDSS sites. [file 12963_2020_230_MOESM3_ESM.docx]

# Additional file 3: Overview of gestational age data collection in Matlab and Bandim HDSS sites

**Collection and calculation of GA in Matlab HDSS**

*Collection:* Matlab HDSS data collectors visit every household once in two months to register vital events and selected maternal and child health information, including contraceptive use, child vaccination and maternal tetanus vaccination [1]. All currently married women aged 15-49 years are also asked whether they missed their last menstruation as an indicator of pregnancy. The data collectors offer a pregnancy test to those who missed it. If the test is positive, the women are asked about the first date of her last menstrual period (LMP), which is then recorded in the record book kept in the women’s households. The data collectors also record this date in the pregnancy outcome registration form after the end of pregnancy. Thus, the recall period for the date of LMP varies by two to three months. However, data collectors in Matlab found approximately one in five of the women absent at home during their routine visit; three quarter of these absent women was found at home during data collectors’ next visit. The recall period for the date of LMP then increases to four to five months for them. For the remaining ~5% of the women, it can be six months or more. Similarly, approximately 80% of the pregnancy outcome dates are recorded within 2-3 months, 15% within 4-5 months and for remaining 5%, it is more than five months.

*Calculation:* GA in weeks = Integer(birth date - LMP date)/7

**Collection and calculation of GA in Bandim HDSS**

*Collection:* In the Bandim HDSS information on GA in months is collected at the time of registering a pregnancy. In the urban area, pregnancies are registered at monthly visits to all houses in the study area, in the rural area pregnancies are registered at six, bi- or monthly visits dependent on the area and year of registration [2]. If a woman was not present when her pregnancy was registered, the question on month of gestation is repeated at a subsequent 6-monthly visit in the rural areas, while no additional attempts to obtain information are made in the urban area. Information on gestational age has been entered for pregnancies registered after May 2013 in the urban area. Like the pregnancies, births are registered at monthly, bi- or six monthly visits. Furthermore, a team of HDSS fieldworkers are present at the national hospital where approximately half of the births in the urban area take place, and here births are registered daily.

*Calculation:* GA in weeks = Integer((((Women reported GAm in completed months on the day of pregnancy registration + 0.5) x30.4) + (delivery date – pregnancy registration date))/7)

**Ultrasound based GA from Matlab hospital**

Gestational age on the day of ultrasound was collected from Matlab hospital for the period of February 2012 to March 2018. Mothers’ HDSS IDs were recorded at ultrasound service. Pregnancy outcomes in HDSS and ultrasound reports at Matlab hospital were linked using the HDSS IDs.

*Use of crown rump length (CRL), bi-parietal diameter (BPD) and femur length (FL) in GA estimation:* GA is estimated by CRL for pregnancies of <14 weeks, and by BPD and FL for pregnancies of ≥14 weeks. We note that Matlab hospital uses *SonoSite M-Turbo* ultrasound equipment. Further details of the algorithm used by the device to estimate GA on the day of ultrasound scan are available at: <https://www.sonosite.com/support/userdoc/M-Turbo_1.8_UG_ENG_P07662-07A_e.pdf?T160=60>

*Who conduct the ultrasound scans?* Trained nurses conduct the ultrasound scans. Trained physicians conduct in cases of the women who are referred as high risk mother, or when the women seem to have complications during her antenatal check-up.

*Quality control of the ultrasound device:* The ultrasound scan data are used for clinical assessment and decisions, and also for research purposes. To ensure the precise diagnosis, ultrasound device troubleshooting is done once a year by biomedical engineers. Image processing and interpretation is independently evaluated two times a year by an expert sinologist.

Calculation of GA at birth: *GA in weeks =Integer[(GA in days on day of ultrasound) + (date of birth from HDSS – date of ultrasound from Matlab hospital)]/7;*

# References

1. Alam N, Ali T, Razzaque A, Rahman M, Zahirul Haq M, Saha SK, Ahmed A, Sarder A, Moinuddin Haider M, Yunus M: **Health and demographic surveillance system (HDSS) in Matlab, Bangladesh.** *International Journal of Epidemiology* 2017, **46:**809-816.

2. Thysen SM, Fernandes M, Benn CS, Aaby P, Fisker AB: **Cohort profile: Bandim Health Project’s (BHP) rural Health and Demographic Surveillance System (HDSS)—a nationally representative HDSS in Guinea-Bissau.** *BMJ Open* 2019, **9:**e028775.
