# Supplementary material for: Gestational age data completeness, quality and validity in population-based surveys: EN-INDEPTH study
Source: Popul Health Metr. 2021 Feb 8;19(Suppl 1):16. doi: 10.1186/s12963-020-00230-3 (PMC7869446; doi:10.1186/s12963-020-00230-3)
Supplement: Supplementary file 4 — Additional file 4. Linking between EN-INDEPTH survey and HDSS data. [file 12963_2020_230_MOESM4_ESM.docx]

# Additional file 4: Linking between EN-INDEPTH survey and HDSS data

EN-INDEPTH survey used women’s HDSS IDs to link between HDSS and survey. Against births of women, there was no such ID embedded in survey to link with HDSS. So, for every woman, we matched birth in survey with HDSS by date of birth. In many cases, the date of birth reported in survey didn’t match exactly with HDSS record. We used probabilistic linking method to match potential pair of records based on probabilistic scores. Pairs with higher scores indicate a better match than pairs with lower scores. We used the *dtalink* package of Stata 15.1 for the probabilistic linking [1]. Steps in the linking process:

***Step 1:*** Separate data files of pregnancy outcomes for survey and HDSS were prepared. Every birth outcome had mother’s common ID as *blocking variable,* and two common linking variables (event date and century month code (CMC) of birth outcome) in both files.

***Step 2:*** Actual event date and CMC wise caliper matching were applied; score ‘0’ was taken as cutoff point; and Stata’s *bestmatch* option was chosen. Score > 30 was considered for matching. The matching status by the probabilistic scores is give below:

| Score | Freq. | Event date difference between Survey and HDSS | Linking variable |
| --- | --- | --- | --- |
| 0.00 | 1,659 | No match (excluded) | CMC of event date |
| 1.00 | 307 | >3 months difference (excluded) | CMC of event date |
| 11.00 | 681 | 3 months difference (excluded) | CMC of event date |
| 31.00 | 2,106 | 1 month difference | CMC of event date |
| 61.00 | 312 | 2 weeks difference | Actual event date |
| 111.00 | 2,215 | 1 week difference | Actual event date |
| 211.00 | 17,606 | No difference | Actual event date |
| Total | 24,886 |  |  |

***Step 3:*** After keeping all the matched pairs, linked dataset was partitioned into two separate data files with individual HDSS and survey information. Both the files had one automatically generated common ID for each matched pair after using probabilistic linking.

***Step 4:*** Final step was to merge them again, but this time using matched ID by 1:1 matching approach of Stata’s built in command *merge*.

# References

1. Kranker K: **dtalink: Faster probabilistic record linking and deduplication methods in Stata for large data files.** *Stata Conference* 2018.
