# Supplementary material for: Gestational age data completeness, quality and validity in population-based surveys: EN-INDEPTH study
Source: Popul Health Metr. 2021 Feb 8;19(Suppl 1):16. doi: 10.1186/s12963-020-00230-3 (PMC7869446; doi:10.1186/s12963-020-00230-3)
Supplement: Supplementary file 5 — Additional file 5. Calculation of survey weights. 5.1: Methods for calculation of survey weights. 5.2: Weighted numbers of livebirths by HDSS sites. [file 12963_2020_230_MOESM5_ESM.docx]

# Additional file 5: Calculation of survey weights

## **Additional file 5.1 Methods for calculation of survey weights**

Questions on gestational age included in section 4 were administered in a sub-sample of women having:

- Last stillbirths occurred on or after 1 January 2012 and recorded in FPH and FBH+ modules
- Last live births occurred on or after 1 January 2012 and ended in neonatal deaths, recorded in FPH and FBH+ modules, and
- Last live births occurred on or after 1 January 2012, and the children survived neonatal death, recorded in FBH+ only

In this paper, we include only live births, and data on stillbirths are excluded from all following analyses, including the calculation of survey weights.

**Sampling weights calculation**

***Step 1:*** Calculate the probability of a woman with any pregnancy outcome (livebirths that survived neonatal period and neonatal deaths) since 1^st^ January 2012 receiving the pregnancy and birth module. The weight was calculated as:

$$\frac{100}{\% of women in 'FPH' and 'FBH+' arm completing pregnancy and birth module}$$

For a woman with a neonatal death since 1^st^ January 2012 the probability of being included is 1, as all women with a neonatal death received these additional questions.

On the other hand, for a woman with a livebirth surviving the neonatal period (from FBH+ only), the probability of receiving the additional questions varied by HDSS site. The calculated weights for five sites are:

Bandim 0.283;

Dabat 0.994;

IgangaMayuge phase I – 0.992, phase II – 0.306;

Kintampo phase I – 0.991, phase II – 0.307;

Matlab 0.296;

***Step 2:*** The weight for each individual pregnancy outcome was calculated as the inverse of the probability of the pregnancy outcome being selected for each record. For example, if a woman had 2 livebirths after 2012, only the last livebirth could be included so the individual sampling weight would be 2/1.

***Step 3:*** The weights calculated under steps 1 and 2 were normalized. First the mean weight of all the selected records was calculated. Then the weights calculated in step 2 were divided by the mean weight to estimate new weights. The mean of new weights is 1.

## **Additional file 5.2 Weighted numbers of livebirths by HDSS sites**

|  | **HDSS sites** | | | | | **All sites** |
| --- | --- | --- | --- | --- | --- | --- |
|  | ***Bandim*** | ***Dabat*** | ***IgangaMayuge*** | ***Kintampo*** | ***Matlab*** |  |
| Number of last live birth in sub-sample (unweighted) | 1,653 | 3,149 | 1,948 | 3,964 | 3,146 | 13,860 |
| Number of last live birth in sub-sample (weighted) | 2,817 | 1,978 | 2,011 | 3,629 | 4,650 | 15,086 |
