# Supplementary material for: Gestational age data completeness, quality and validity in population-based surveys: EN-INDEPTH study
Source: Popul Health Metr. 2021 Feb 8;19(Suppl 1):16. doi: 10.1186/s12963-020-00230-3 (PMC7869446; doi:10.1186/s12963-020-00230-3)
Supplement: Supplementary file 6 — Additional file 6. Qualitative methods for Focus Group Discussions in the EN-INDEPTH study. [file 12963_2020_230_MOESM6_ESM.docx]

# Additional file 6: Qualitative methods for Focus Group Discussions in the EN-INDEPTH study

To identify community perceptions, practices, and barriers to reporting outcomes, and how these contribute to understanding of the measurement of any outcome in population-based surveys, 34 Focus Group Discussions (FGDs) were undertaken. The number of FGDs undertaken was similar across the sites: Bandim (n)=6, Dabat (n)=5, IgangaMayuge (n)=7, Matlab (n)=8 and Kintampo (n)=8.

The FGDs explored women’s (respondents), survey interviewers’ and HDSS data collectors’ experiences with the EN-INDEPTH survey and/or the HDSS data collection process, as well as attitudes, knowledge, and practices around reporting and disclosure of pregnancy and adverse pregnancy outcomes and associated enablers and challenges.

A common training and implementation manual was developed for use across the sites. Interviews were held in the most commonly spoken local language (or English) by moderators and note takers who had skills in qualitative data collection methods. Data were transcribed locally and analysed by HDSS study teams, with at least two analysts participating in coding.
