# Supplementary material for: Gestational age data completeness, quality and validity in population-based surveys: EN-INDEPTH study
Source: Popul Health Metr. 2021 Feb 8;19(Suppl 1):16. doi: 10.1186/s12963-020-00230-3 (PMC7869446; doi:10.1186/s12963-020-00230-3)
Supplement: Supplementary file 7 — Additional file 7. Results: Additional details. 7.1: Gestational age capture in weeks in the EN-INDEPTH survey. 7.1A: Logit estimates of adjusted ORs and 95% confidence interval of responding to GA in weeks. 7.1B: Matching of GA months with GA weeks, EN-INDEPTH survey. 7.1C: Gestational age distribution by religion, Matlab site, EN-INDEPTH survey in last five years. 7.2: Comparison of GA weeks between survey, HDSS and early pregnancy ultrasound, Matlab site. 7.2A: GA weeks in last five years by HDSS, early pregnancy ultrasound and five years prior to EN-INDEPTH survey. 7.2B: GA weeks for livebirths by HDSS, early pregnancy ultrasound and EN-INDEPTH survey in last five years. 7.2C: Early pregnancy ultrasound versus EN-INDEPTH survey and HDSS data in last five years by ultrasound timing. 7.2D: Adjusted relative risk ratios for over- and under-reporting of GA weeks, survey and HDSS versus ultrasound. 7.3: Community perceptions, practices and barriers to reporting GA, EN-INDEPTH study (five sites). 7.4: Comparison of preterm birth rates in EN-INDEPTH study to external data sources. [file 12963_2020_230_MOESM7_ESM.docx]

# Additional file 7: Results: Additional details

## **Additional file 7.1: Gestational age capture in weeks in the EN-INDEPTH survey (five sites, n=15,086)**

### Additional file 7.1A: Logit estimates of adjusted ORs and 95% confidence interval of responding to GA in weeks (n= 13,962)

| Characteristics of women | Adjusted odds ratios  [95% confidence interval] | | | | |
| --- | --- | --- | --- | --- | --- |
|  | Bandim | Dabat | IgangaMayuge | Kintampo | Matlab |
| Women’s age  (ref: 20-24 years) |  |  |  |  |  |
| 15-19 | 0.84  [0.18, 3.98] | 1.56  [0.7, 3.46] | a | 1.43  [0.87, 2.34] | a |
| 25-29 | 1.43  [0.61, 3.35] | 1.52  [0.96, 2.43] | 0.62  [0.19, 2.02] | 1.10  [0.81, 1.49] | 1.11  [0.17, 7.38] |
| 30+ | 4.52  [1.93, 10.61] | 1.16  [0.7, 1.91] | 1.32  [0.3, 5.89] | 1.15  [0.83, 1.57] | 0.98  [0.04, 26.23] |
| Children ever born  (ref: 1) |  |  |  |  |  |
| 2 | 0.59  [0.29, 1.19] | 1.05  [0.66, 1.67] | 1.84  [0.48, 7.03] | 1.08  [0.81, 1.46] | 1.55  [0.20, 12.16] |
| 3+ | 0.37  [0.19, 0.73] | 0.91  [0.54, 1.51] | 4.14  [1.14, 15.07] | 0.81  [0.59, 1.1] | 2.33  [0.05, 98.56] |
| Year of schooling  (ref: Never attended school) |  |  |  |  |  |
| 0-5 | 2.00  [0.90, 4.47] | 1.00  [0.66, 1.52] | 1.96  [0.46, 8.36] | 1.15  [0.92, 1.44] | 18.6  [2.05, 168.69] |
| ≥6 | 1.61  [0.62, 4.19] | 1.05  [0.68, 1.62] | 1.37  [0.27, 6.96] | 1.02 [0.82, 1.27] | 6.61  [1.19, 36.61] |
| Wealth quintile  (ref: Lowest) |  |  |  |  |  |
| Second | 0.68  [0.36, 1.27] | 0.81  [0.44, 1.48] | 2.20  [0.55, 8.78] | 0.62  [0.47, 0.8] | 0.86  [0.14, 5.42] |
| Middle | 0.53  [0.26, 1.07] | 0.85  [0.46, 1.54] | 1.58  [0.53, 4.74] | 0.51  [0.38, 0.67] | 0.77  [0.08, 7.65] |
| Fourth | 0.62  [0.28, 1.38] | 0.33  [0.19, 0.58] | 1.46  [0.46, 4.62] | 0.54  [0.41, 0.73] | 0.32  [0.07, 1.47] |
| Highest | 0.07  [0.02, 0.31] | 0.17  [0.09, 0.30] | 1.44  [0.46, 4.45] | 0.74  [0.55, 1.00] | a |
| Religion  (ref: Muslim) |  |  |  |  |  |
| Christian | 1.89  [0.99, 3.61] | 1.03  [0.60, 1.76] | 1.09  [0.52, 2.28] | 0.91  [0.76, 1.10] | b |
| Other or none | 1.26  [0.53, 3.00] | a | a | 0.64  [0.43, 0.95] | 2.11  [0.22, 20.31] |
| ANC visits  (ref: <4) |  |  |  |  |  |
| 4+ | 1.2  [0.52, 2.79] | 0.59  [0.43, 0.81] | 2.79  [1.18, 6.60] | 0.94  [0.74, 1.20] | 0.83  [0.08, 8.06] |
| DK/missing | 0.87  [0.30, 2.57] | 0.99  [0.60, 1.63] | 0.22  [0.04, 1.30] | 0.18  [0.13, 0.24] | 1.38  [0.16, 11.55] |
| Place of delivery  (ref: Home) |  |  |  |  |  |
| Facility | 1.77  [0.36, 8.84] | 0.77  [0.28, 2.11] | 2.05  [0.40, 10.41] | 0.86  [0.43, 1.73] | 0.53  [0.05, 5.51] |
| Mode of delivery  (ref: Normal) |  |  |  |  |  |
| C-section | 1.90  [0.93, 3.87] | 0.97  [0.54, 1.73] | 0.85  [0.16, 4.48] | 0.98  [0.71, 1.37] | 6.48  [0.60, 70.45] |
| Missing | 1.13  [0.26, 4.91] | 0.91  [0.34, 2.44] | 0.39  [0.09, 1.78] | 0.68  [0.34, 1.37] | 1.65  [0.11, 25.56] |
| Constant | 0.02  [0.00, 0.14] | 29.72  [8.06, 109.55] | 3.27  [0.19, 55.72] | 3.19  [1.42, 7.16] | 29.79  [1.52, 585.5] |
| Observations | 2,817 | 1,979 | 1,930 | 3,629 | 3,607 |
| a: Too few responses on GA in weeks; b: none of the respondents are Christian. | | | | | |

### Additional file 7.1B: Matching of GA months with GA weeks, EN-INDEPTH survey (five sites, n=9,811)

|  |  | Gestational age in weeks (%) | | | | | | | | | | | | | | | | | | | | | | | | | |  |
| --- | --- | --- | --- | --- | --- | --- | --- | --- | --- | --- | --- | --- | --- | --- | --- | --- | --- | --- | --- | --- | --- | --- | --- | --- | --- | --- | --- | --- |
|  | Site |  | 22 | 23 | 24 | 25 | 26 | 27 | 28 | 29 | 30 | 31 | 32 | 33 | 34 | 35 | 36 | 37 | 38 | 39 | 40 | 41 | 42 | 43 | 44 | 45 | Total | n |
| Gestational age in months | Bandim | 6 | - | - | - | - | - | - | - | - | - | - | - | - | - | - | - | - | - | - | - | - | - | - | - | - | - | 0 |
|  |  | 7 | 0.0 | 0.0 | 0.0 | 0.0 | 0.0 | 0.0 | 0.0 | 0.0 | 0.0 | 0.0 | 0.0 | 0.0 | 0.0 | 0.0 | 100.0 | 0.0 | 0.0 | 0.0 | 0.0 | 0.0 | 0.0 | 0.0 | 0.0 | 0.0 | 100.0 | 1 |
|  |  | 8 | 0.0 | 0.0 | 0.0 | 0.0 | 0.0 | 0.0 | 0.0 | 0.0 | 0.0 | 0.0 | 0.0 | 0.0 | 50.0 | 0.0 | 50.0 | 0.0 | 0.0 | 0.0 | 0.0 | 0.0 | 0.0 | 0.0 | 0.0 | 0.0 | 100.0 | 2 |
|  |  | 9 | 0.0 | 0.0 | 0.0 | 0.0 | 0.0 | 0.0 | 1.3 | 0.0 | 0.0 | 0.0 | 6.3 | 0.0 | 0.0 | 0.0 | 53.8 | 12.5 | 11.3 | 0.0 | 8.8 | 2.5 | 2.5 | 0.0 | 0.0 | 1.3 | 100.0 | 80 |
|  |  | 10 | 0.0 | 0.0 | 0.0 | 0.0 | 0.0 | 0.0 | 0.0 | 0.0 | 0.0 | 0.0 | 0.0 | 0.0 | 0.0 | 0.0 | 0.0 | 100.0 | 0.0 | 0.0 | 0.0 | 0.0 | 0.0 | 0.0 | 0.0 | 0.0 | 100.0 | 1 |
|  |  | 11 | - | - | - | - | - | - | - | - | - | - | - | - | - | - | - | - | - | - | - | - | - | - | - | - | - | 0 |
|  | Dabat | 6 | - | - | - | - | - | - | - | - | - | - | - | - | - | - | - | - | - | - | - | - | - | - | - | - | - | 0 |
|  |  | 7 | 0.0 | 0.0 | 5.0 | 0.0 | 0.0 | 0.0 | 65.0 | 5.0 | 0.0 | 0.0 | 5.0 | 0.0 | 5.0 | 0.0 | 15.0 | 0.0 | 0.0 | 0.0 | 0.0 | 0.0 | 0.0 | 0.0 | 0.0 | 0.0 | 100.0 | 20 |
|  |  | 8 | 0.0 | 0.0 | 0.0 | 0.0 | 0.0 | 0.0 | 0.0 | 0.0 | 0.0 | 0.0 | 77.8 | 0.0 | 11.1 | 0.0 | 0.0 | 11.1 | 0.0 | 0.0 | 0.0 | 0.0 | 0.0 | 0.0 | 0.0 | 0.0 | 100.0 | 9 |
|  |  | 9 | 0.0 | 0.0 | 0.0 | 0.0 | 0.0 | 0.0 | 0.0 | 0.0 | 0.0 | 0.0 | 0.3 | 0.0 | 0.1 | 0.1 | 96.2 | 1.5 | 1.0 | 0.2 | 0.1 | 0.4 | 0.1 | 0.0 | 0.0 | 0.0 | 100.0 | 2815 |
|  |  | 10 | 0.0 | 0.0 | 0.0 | 0.0 | 0.0 | 0.0 | 0.0 | 0.0 | 0.0 | 0.0 | 0.0 | 0.0 | 0.0 | 0.0 | 0.0 | 0.0 | 0.0 | 0.0 | 100 | 0.0 | 0.0 | 0.0 | 0.0 | 0.0 | 100.0 | 3 |
|  |  | 11 | 0.0 | 0.0 | 0.0 | 0.0 | 0.0 | 0.0 | 0.0 | 0.0 | 0.0 | 0.0 | 0.0 | 0.0 | 0.0 | 0.0 | 0.0 | 0.0 | 0.0 | 0.0 | 0.0 | 0.0 | 0.0 | 0.0 | 100 | 0.0 | 100.0 | 1 |
|  | IgangaMayuge | 6 | 0.0 | 0.0 | 100 | 0.0 | 0.0 | 0.0 | 0.0 | 0.0 | 0.0 | 0.0 | 0.0 | 0.0 | 0.0 | 0.0 | 0.0 | 0.0 | 0.0 | 0.0 | 0.0 | 0.0 | 0.0 | 0.0 | 0.0 | 0.0 | 100.0 | 8 |
|  |  | 7 | 0.0 | 0.0 | 0.0 | 7.7 | 0.0 | 0.0 | 61.5 | 0.0 | 0.0 | 0.0 | 7.7 | 0.0 | 0.0 | 0.0 | 23.1 | 0.0 | 0.0 | 0.0 | 0.0 | 0.0 | 0.0 | 0.0 | 0.0 | 0.0 | 100.0 | 13 |
|  |  | 8 | 0.0 | 0.0 | 0.0 | 0.0 | 1.8 | 0.0 | 3.6 | 0.0 | 0.0 | 0.0 | 60.0 | 1.8 | 18.2 | 5.5 | 1.8 | 1.8 | 5.5 | 0.0 | 0.0 | 0.0 | 0.0 | 0.0 | 0.0 | 0.0 | 100.0 | 55 |
|  |  | 9 | 0.0 | 0.0 | 0.1 | 0.1 | 0.0 | 0.0 | 0.3 | 0.0 | 0.0 | 0.0 | 4.8 | 0.1 | 0.1 | 0.2 | 68.4 | 6.5 | 15.6 | 1.0 | 0.8 | 0.0 | 1.7 | 0.1 | 0.1 | 0.0 | 100.0 | 1733 |
|  |  | 10 | 0.0 | 0.0 | 0.0 | 0.0 | 0.0 | 0.0 | 0.0 | 0.0 | 0.0 | 0.0 | 0.0 | 0.0 | 0.0 | 0.0 | 8.9 | 5.4 | 0.0 | 0.0 | 73.2 | 1.8 | 8.9 | 0.0 | 1.8 | 0.0 | 100.0 | 56 |
|  |  | 11 | 0.0 | 0.0 | 0.0 | 0.0 | 0.0 | 0.0 | 0.0 | 0.0 | 0.0 | 0.0 | 0.0 | 0.0 | 0.0 | 0.0 | 9.1 | 0.0 | 0.0 | 0.0 | 9.1 | 0.0 | 9.1 | 0.0 | 72.7 | 0.0 | 100.0 | 11 |
|  | Kintampo | 6 | 0.0 | 0.0 | 50.0 | 0.0 | 0.0 | 0.0 | 0.0 | 0.0 | 0.0 | 0.0 | 0.0 | 0.0 | 0.0 | 0.0 | 50.0 | 0.0 | 0.0 | 0.0 | 0.0 | 0.0 | 0.0 | 0.0 | 0.0 | 0.0 | 100.0 | 2 |
|  |  | 7 | 0.0 | 0.0 | 0.0 | 0.0 | 0.0 | 2.9 | 74.3 | 2.9 | 2.9 | 0.0 | 8.6 | 0.0 | 0.0 | 0.0 | 5.7 | 0.0 | 2.9 | 0.0 | 0.0 | 0.0 | 0.0 | 0.0 | 0.0 | 0.0 | 100.0 | 35 |
|  |  | 8 | 0.0 | 0.0 | 0.0 | 0.0 | 0.0 | 0.0 | 0.0 | 0.0 | 6.3 | 0.0 | 62.5 | 0.0 | 31.3 | 0.0 | 0.0 | 0.0 | 0.0 | 0.0 | 0.0 | 0.0 | 0.0 | 0.0 | 0.0 | 0.0 | 100.0 | 16 |
|  |  | 9 | 0.0 | 0.0 | 0.1 | 0.0 | 0.0 | 0.0 | 0.8 | 0.0 | 0.4 | 0.1 | 1.2 | 0.3 | 1.9 | 1.0 | 73.2 | 7.1 | 9.5 | 2.2 | 1.4 | 0.4 | 0.3 | 0.0 | 0.1 | 0.0 | 100.0 | 1945 |
|  |  | 10 | 0.0 | 0.0 | 0.0 | 0.0 | 0.0 | 0.0 | 0.0 | 0.0 | 0.0 | 0.0 | 0.6 | 0.6 | 0.6 | 0.0 | 11.2 | 3.0 | 8.9 | 8.9 | 54.4 | 6.5 | 4.1 | 1.2 | 0.0 | 0.0 | 100.0 | 169 |
|  |  | 11 | 0.0 | 0.0 | 0.0 | 0.0 | 0.0 | 0.0 | 0.0 | 0.0 | 0.0 | 0.0 | 0.0 | 0.0 | 4.0 | 0.0 | 12.0 | 4.0 | 4.0 | 8.0 | 0.0 | 4.0 | 12.0 | 0.0 | 48.0 | 4.0 | 100.0 | 25 |
|  | Matlab | 6 | 0.0 | 0.0 | 8.0 | 12.0 | 12.0 | 28.0 | 16.0 | 20.0 | 4.0 | 0.0 | 0.0 | 0.0 | 0.0 | 0.0 | 0.0 | 0.0 | 0.0 | 0.0 | 0.0 | 0.0 | 0.0 | 0.0 | 0.0 | 0.0 | 100.0 | 25 |
|  |  | 7 | 0.0 | 0.0 | 0.0 | 0.0 | 0.0 | 0.0 | 15.1 | 15.1 | 27.9 | 16.3 | 12.8 | 8.1 | 1.2 | 2.3 | 0.0 | 1.2 | 0.0 | 0.0 | 0.0 | 0.0 | 0.0 | 0.0 | 0.0 | 0.0 | 100.0 | 86 |
|  |  | 8 | 0.0 | 0.0 | 0.0 | 0.0 | 0.0 | 0.0 | 0.4 | 0.0 | 0.4 | 0.0 | 3.8 | 3.8 | 22.6 | 14.7 | 21.4 | 22.2 | 9.7 | 0.6 | 0.0 | 0.2 | 0.2 | 0.0 | 0.0 | 0.0 | 100.0 | 496 |
|  |  | 9 | 0.0 | 0.0 | 0.0 | 0.0 | 0.0 | 0.1 | 0.1 | 0.0 | 0.0 | 0.0 | 0.1 | 0.0 | 0.2 | 0.6 | 8.5 | 4.0 | 51.5 | 20.6 | 11.3 | 1.7 | 1.4 | 0.0 | 0.0 | 0.0 | 100.0 | 1878 |
|  |  | 10 | 0.3 | 0.0 | 0.0 | 0.0 | 0.0 | 0.0 | 0.0 | 0.0 | 0.0 | 0.0 | 0.3 | 0.0 | 0.0 | 0.0 | 1.8 | 0.9 | 3.7 | 5.8 | 25.2 | 2.5 | 58.0 | 1.2 | 0.3 | 0.0 | 100.0 | 326 |
|  |  | 11 | - | - | - | - | - | - | - | - | - | - | - | - | - | - | - | - | - | - | - | - | - | - | - | - | - | 0 |
| Note: Gray cells represent values GAw = 4xGAm; yellow cells represent values GAw = 4xGAm+2 for Matlab site only.  GAw≤21 and GAm≤21 excluded. | | | | | | | | | | | | | | | | | | | | | | | | | | | | |

### Additional file 7.1C: Gestational age distribution by religion, Matlab site, EN-INDEPTH survey in last five years

|  |  |
| --- | --- |
| ***Note:*** (GAw (n=4,178 ), GAm (n=20,891)). GAm (survey) ≤5 and GAw (HDSS) ≤21 are excluded | |

## **Additional file 7.2: Comparison of GA weeks between survey, HDSS and early pregnancy ultrasound, Matlab site**

### Additional file 7.2A: GA weeks in last five years by HDSS, early pregnancy ultrasound and five years prior to EN-INDEPTH survey

(Ultrasound(n=5,040), HDSS(n=23,750), survey(n=2,907))

### Additional file 7.2B: GA weeks for livebirths by HDSS, early pregnancy ultrasound and EN-INDEPTH survey in last five years

Note: For the unmatched cases. (Ultrasound(n=9,488), HDSS(n=22,671), Survey(n=1,841))

### Additional file 7.2C: Early pregnancy ultrasound versus EN-INDEPTH survey and HDSS data in last five years by ultrasound timing

Preterm birth rates

Ultrasound: 16%

HDSS: 14%

Survey: 24%

Preterm birth rates

Ultrasound: 13%

HDSS: 11%

Survey: 20%

Note: For the cases that matched in the three data sources (Matlab only, n=481)

### Additional file 7.2D: Adjusted relative risk ratios for over- and under-reporting of GA weeks, survey and HDSS versus ultrasound

| Characteristics of women | Adjusted relative risk ratios [95% confidence interval] | | | |
| --- | --- | --- | --- | --- |
|  | Over-reporting  (ref: equal-reporting) | | Under-reporting  (ref: equal-reporting) | |
|  | HDSS | Survey | HDSS | Survey |
| Women’s age in years *(ref: 20-24)* |  |  |  |  |
| 15-19 | 0.89 [0.32, 2.47] | 1.38 [0.39, 4.84] | 0.51 [0.14, 1.81] | 1.62 [0.49, 5.41] |
| 25-29 | 0.89 [0.46, 1.71] | 1.05 [0.48, 2.31] | 1.39 [0.70, 2.74] | 1.00 [0.48, 2.09] |
| 30-34 | 1.45 [0.66, 3.17] | 1.18 [0.48, 2.92] | 1.97 [0.88, 4.44] | 0.58 [0.24, 1.39] |
| ≥35 | 1.03 [0.41, 2.63] | 0.71 [0.24, 2.08] | 1.47 [0.56, 3.86] | 0.48 [0.17, 1.34] |
| Children ever born  *(ref: 1)* |  |  |  |  |
| 2 | 0.91 [0.48, 1.71] | 1.15 [0.55, 2.40] | 0.87 [0.45, 1.71] | 1.67 [0.82, 3.39] |
| ≥3 | 0.98 [0.43, 2.25] | 0.86 [0.33, 2.22] | 1.27 [0.55, 2.96] | 2.01 [0.81, 4.96] |
| Year of schooling  *(ref: 6-9)* |  |  |  |  |
| Never attended school | 0.19 [0.04, 1.02] | 6.87 [0.78, 60.34] | 0.47 [0.12, 1.88] | 2.62 [0.27, 25.77] |
| 0-5 | 3.59 [1.65, 7.84] | 1.51 [0.68, 3.32] | 2.55 [1.13, 5.72] | 0.92 [0.42, 2.01] |
| ≥10 | 0.90 [0.43, 1.87] | 1.13 [0.47, 2.73] | 1.36 [0.66, 2.79] | 1.78 [0.80, 3.99] |
| Wealth quintile  *(ref: Lowest)* |  |  |  |  |
| Second | 0.47 [0.22, 1.02] | 1.10 [0.44, 2.74] | 1.09 [0.48, 2.47] | 2.41 [0.99, 5.88] |
| Middle | 0.44 [0.2, 0.96] | 0.83 [0.34, 2.05] | 0.97 [0.42, 2.24] | 2.02 [0.84, 4.83] |
| Forth | 0.45 [0.21, 0.99] | 0.62 [0.26, 1.49] | 1.24 [0.54, 2.88] | 1.24 [0.52, 2.93] |
| Highest | 0.48 [0.22, 1.06] | 0.48 [0.20, 1.17] | 0.96 [0.41, 2.28] | 1.25 [0.53, 2.97] |
| Religion  *(ref: Muslim)* |  |  |  |  |
| Non-Muslim | 2.03 [0.91, 4.53] | 0.56 [0.24, 1.33] | 3.17 [1.45, 6.93] | 0.91 [0.42, 1.97] |
| TV watching *(ref: At least once a week)* |  |  |  |  |
| Less than once a week | 0.65 [0.28, 1.54] | 0.67 [0.24, 1.89] | 0.71 [0.28, 1.77] | 1.15 [0.44, 2.99] |
| Not at all | 0.86 [0.49, 1.48] | 0.58 [0.31, 1.11] | 1.52 [0.87, 2.63] | 1.09 [0.60, 1.99] |
| ANC visits *(ref: 4+)* |  |  |  |  |
| <4 | 0.46 [0.18, 1.18] | 1.63 [0.48, 5.49] | 0.64 [0.27, 1.55] | 2.31 [0.74, 7.24] |
| Don't Know | 1.16 [0.06, 21.38] | - | 1.58 [0.09, 28.82] | 0.98 [0.08, 12.00] |
| Missing | 1.71 [0.83, 3.53] | 3.88 [1.39, 10.87] | 0.87 [0.38, 1.99] | 1.73 [0.61, 4.95] |
| Place of delivery  *(ref: Facility)* |  |  |  |  |
| Home | 1.14 [0.49, 2.69] | 1.46 [0.54, 3.95] | 0.58 [0.22, 1.54] | 0.76 [0.28, 2.06] |
| Recall period in years *(ref: <1)* |  |  |  |  |
| 1-2 | 1.51 [0.78, 2.93] | 0.83 [0.40, 1.72] | 1.13 [0.57, 2.24] | 0.73 [0.36, 1.45] |
| 3-5 | 1.03 [0.60, 1.76] | 1.24 [0.66, 2.34] | 0.73 [0.42, 1.26] | 1.28 [0.70, 2.33] |
| Constant | 1.34 [0.59, 3.07] | 2.15 [0.84, 5.53] | 0.43 [0.17, 1.09] | 1 [0.39, 2.53] |

Note: Ultrasound at ≤23 weeks’ gestation. N=481, Matlab site

## **Additional file 7.3: Community perceptions, practices and barriers to reporting GA, EN-INDEPTH study (five sites)**

| Theme | Sub-Theme | Reported by women, interviewers or both | Site(s) | Potential implications for measurement in population-based surveys |
| --- | --- | --- | --- | --- |
| Perception | 1. Importance to know GA | 1. Both | 1. All sites | - As women perceive different importance to track GA in pregnancy, they tend to count which helps to report GA - Collecting GA from women unaware of importance of GA, not having ANC and delivering at home needs extra care - Women unaware of GA may be unable to report. This portion of women is expected to be low. |
|  | 1.1. Helps in birth planning and preparation | 1.1. Both | 1.1. All sites |  |
|  | 1.2. Helps to inform GA to providers during ANC and in care seeking for pregnancy complications | 1.2. Women | 1.2. Dabat, Kintampo, Matlab |  |
|  | 1.3. Facilitate to get child’s father’s companion at ANC and delivery | 1.3. Women | 1.3. IgangaMayuge |  |
|  | 1.4. So that the biological father can’t deny impregnating her | 1.4. Women | 1.4. IgangaMayuge |  |
|  | 2. Few women are completely unaware of GA | 2. Interviewers | 2. IgangaMayuge, Kintampo, Matlab |  |
|  | 3. Collection of GA data is difficult | 3. Interviewers | 3. All sites |  |
| Practice | 1. Women count GA in months, generally not in weeks | 1. Both | 1. All sites | - Existing practice helps women report GA in months, not in weeks. However, counted days in reported months may vary as different practices are found like missed period and religious practice - Recalling other key events to remember LMP date may be useful to capture GA - Receiving ANC is an enabler in GA reporting |
|  | 2. Many women count GA by missed periods | 2. Both | 2. All sites |  |
|  | 2.1. Some start counting 1 month from first missed period | 3.1. Both | 2.1. All sites |  |
|  | 2.2. Some consider missed period as the start of pregnancy | 3.2. Both | 2.2. All sites |  |
|  | 4. GA counting varies by religious practice | 4. Interviewers | 4. Matlab |  |
|  | 5. Few recall LMP dates by recalling key events in the calendar, e.g., religious holidays, crop harvesting month, etc. | 5. Interviewers | 5. Dabat |  |
|  | 6. ANC providers counting GA | 1. Both | 1. All sites |  |
|  | 6.1. Women not receiving ANC find GA difficult to report | 6.1. Interviewers | 6.1. Bandim |  |
| Barriers |  |  |  | - Needs careful probing to collect GA from women low education and young age. - Method failure of hormonal method or conceiving just after stopping hormonal methods makes GA reporting difficult as menstrual cycle might be irregular due to the methods use. - Confidentiality assuring rapport building may encourage women in fear of others criticism and witchcraft to report GA - Orientation of data collectors with religious beliefs, important socio-cultural and religious, different crop harvesting time, etc. may be useful. |
|  | 1. Women don’t record LMP date, many can’t remember. | 1.1. Interviewers | 1.1. IgangaMayuge, Kintampo, Matlab |  |
|  | 1.2. Many are reluctant to remember LMP date | 1.2. Interviewers | 1.2. Bandim |  |
|  | 2. Not possible to report LMP for women who had conceived before menstruation recurrence after last birth | 2. Interviewers | 2. Matlab |  |
|  | 3. Difficult for women using hormonal contraceptives (due to irregular menstrual cycle) | 3. Interviewers | 3. Dabat, Matlab |  |
|  | 4. Younger women find GA reporting difficult | 4. Interviewers | 4.. Bandim |  |
|  | 5. Women believing in witchcraft are unwilling to report GA | 5. Interviewers | 5. Bandim |  |
|  | 6. Poor record keeping tendency, e.g., not preserving maternity cards | 6. Interviewers | 6. IgangaMayuge, Kintampo |  |
|  | 7. Low education affects GA reporting | 7. Interviewers | 7. Dabat, IgangaMayue, Kintampo, Matlab |  |
|  | 8. Social stigma like fearing criticism of neighbour discourage women to report GA | 8. Interviewers | 8. Bandim |  |

## **Additional file 7.4: Comparison of preterm birth rates in EN-INDEPTH study to external data sources**

| Sources of preterm birth rates  (2012-2018) | Bandim  (Guinea-Bissau) | Dabat  (Ethiopia) | IgangaMayuge  (Uganda) | Kintampo  (Ghana) | Matlab  (Bangladesh) |
| --- | --- | --- | --- | --- | --- |
| GAm EN-INDEPTH survey | 1.7 | 0.8 | 3.0 | 1.4 | 17.0 |
| GAw EN-INDEPTH survey | 59.5 | 96.6 | 69.4 | 71.5 | 20.9 |
| ‘Born before expected’ and ‘weeks born before expected’ EN-INDEPTH survey | 1.0 | 0.7 | 2.8 | 1.7 | 7.7 |
| Routine HDSS | 30.9 | NA | NA | NA | 11.4 |
| Early pregnancy ultrasound (<24 weeks) | - | - | - | - | 12.4 |
| National estimates based on DHS (year, period of recall) | NA | NA | NA | NA | NA |
| National estimates based on MICS survey (year, period of recall) | NA | NA | NA | NA | NA |
| National UN country estimates 2014 [1] | 12.0 | 12.0 | 6.6 | 12.0 | 19.2 |

# References

1. World Health Organisation: **Global preterm birth estimates.** 2018. <http://ptb.srhr.org/> [Accessed June 2020].
