# Supplementary material for: Gestational age data completeness, quality and validity in population-based surveys: EN-INDEPTH study
Source: Popul Health Metr. 2021 Feb 8;19(Suppl 1):16. doi: 10.1186/s12963-020-00230-3 (PMC7869446; doi:10.1186/s12963-020-00230-3)
Supplement: Supplementary file 8 — Additional file 8. Proposed revised questions to capture gestational age. [file 12963_2020_230_MOESM8_ESM.docx]

# Additional file 8: Proposed revised survey questions to capture gestational age

| Sl.# | Questions | Answer | Skip |
| --- | --- | --- | --- |
| GA1 | Did you have ultrasound when you were pregnant with xxx? | Yes 1  No 2🡺 | GA4 |
| GA2 | Can you show me the report? | Yes 1  No 2🡺 | GA4 |
| GA3 | a. Date of ultrasound?  b. Gestational age reported?  c. Age of fetus reported?  *[Notice carefully what was recorded - GA or fetus age]* | a. Day  Month  Year  b.  weeks  days  c.  weeks  days  *[Record ‘a’ and ‘b’, or ‘a’ and ‘c’]* |  |
| GA4 | Do you have any ANC card, maternity card or other health card? | ANC card observed Yes No  Maternity card observed Yes No  Other health card observed Yes No  One of the answers is “Yes” 🡺  All of the answers are “No” 🡺 | GA5  GA12 |
| GA5 | Has the card information on LMP date? | Yes 1  No 2🡺 | GA10 |
| GA6 | Record: | LMP date D M Y  First visit date D M Y |  |
| GA10 | Has the card information on gestational/fetus age? | Gestational age Yes No  Fetus age Yes No  One of the answers is “Yes” 🡺  All of the answers are “No” 🡺 | GA11  GA12 |
| GA11 | Record: | The age  weeks  days  1^st^ visit date D M Y |  |
| GA12 | When xxx was born, how far along you were with your pregnancy?  *[Let respondent answer in her way. Don’t prompt to answer in months/weeks. ]* | *Write exact answer in space below. then fill in* ‘a’, ‘b’ *and* ‘c’  a. Months  *[If reported in*  b. Weeks  *weeks and/or days]* 🡺  c. Days | GA16 |
| GA13 | You have mentioned pregnancy length in months. How did you count a month?  *[Check the respective box]* | a. Calendar months 🡺  b. 30 days months 🡺  c. 28 days months 🡺  d. Menstruation cycle as month 🡺  e. Other (e.g., religious/cultural) 🡺 | GA16  GA16  GA16  GA14  GA15 |
| GA14 | What is the usual gap between your two consecutive menstruations? | days 🡺 | GA16 |
| GA15 | How many days do you count against the type of month you have reported? | days |  |
| GA16 | Necessary data to calculate GA are collected above based on different sources (ultrasound, card, or recall). We GA in days can be calculated from the data. | |  |
| Note: The above draft question set has been developed to collect GA data for recent births in the last 5 years at the time of household survey based on the learning around GA measurement from the EN-INDEPTH study. The applicability of questions/phrases/words used will be assessed through piloting. A user manual will be developed to enable implementation of the final question set. The responses will be used to calculate GA – using ultrasound data if available and ultrasound at <24 weeks, otherwise health card data will be used when available; finally if no ultrasound or card data available woman-directly reported data will be used. | | | |
